# Gene-expression memory-based prediction of cell lineages from scRNA-seq datasets

**DOI:** 10.1038/s41467-024-47158-y

**Published:** 2024-03-29

**Authors:** A. S. Eisele, M. Tarbier, A. A. Dormann, V. Pelechano, D. M. Suter

**Affiliations:** 1 Ecole Polytechnique Fédérale de Lausanne, School of Life Sciences, Institute of Bioengineering, Lausanne, Switzerland; 2 Science for Life Laboratory, Department of Microbiology, Tumor and Cell Biology, Karolinska Institute, Solna, Sweden

**Keywords:** Computational biology and bioinformatics, Epigenetic memory, Gene expression profiling, Computational models, Gene ontology

## Abstract

Assigning single cell transcriptomes to cellular lineage trees by lineage tracing has transformed our understanding of differentiation during development, regeneration, and disease. However, lineage tracing is technically demanding, often restricted in time-resolution, and most scRNA-seq datasets are devoid of lineage information. Here we introduce Gene Expression Memory-based Lineage Inference (GEMLI), a computational tool allowing to robustly identify small to medium-sized cell lineages solely from scRNA-seq datasets. GEMLI allows to study heritable gene expression, to discriminate symmetric and asymmetric cell fate decisions and to reconstruct individual multicellular structures from pooled scRNA-seq datasets. In human breast cancer biopsies, GEMLI reveals previously unknown gene expression changes at the onset of cancer invasiveness. The universal applicability of GEMLI allows studying the role of small cell lineages in a wide range of physiological and pathological contexts, notably in vivo. GEMLI is available as an R package on GitHub (https://github.com/UPSUTER/GEMLI).

## Abstract (web summary)

Combining experimental lineage tracing with single cell transcriptomics is technically demanding. Here, authors present GEMLI, a computational tool to annotate cell lineages in single cell RNA sequencing data solely based on gene expression.

## Introduction

In multicellular organisms, each cell belongs to a lineage tree that determines its relatedness to other cells. Cell lineage relationships are of fundamental biological relevance as they directly impact a broad range of cellular behaviors, such as cancer resistance to drugs^1–7^, disease onset^3,8^, and differentiation in development, homeostasis, and regeneration^9–15^. Dissecting early gene expression changes that occur during cell fate switches is essential to understand the mechanistic bases of these processes. This requires to focus on small cell lineages in which cell fate decisions occur. While cellular characterization by scRNA-seq provides rich molecular information, it is intrinsically devoid of lineage information. Therefore, there is considerable interest in identifying cell lineages in scRNA-seq datasets.

The most widely used technique to assign individual transcriptomes to given lineages is cellular barcoding, which involves the introduction of heritable, expressed DNA barcodes in individual cells that can be retrieved in scRNA-seq data^7,9,12,16–19^. Other approaches involve hand-picking of related cells visualized by microscopy or microfluidic devices to compartmentalize cell lineages before scRNA-seq^20–25^. However, all these techniques suffer from important technical limitations such as the requirement for dedicated devices, extensive cell handling and/or culture, or genetic engineering.

Natural genetic marks such as mitochondrial mutations, copy number variants, and somatic mutations have been used to identify related cells in scRNA-seq datasets, mainly after enrichment for desired transcripts^26–32^. Still, the scarcity of spontaneously occurring genetic marks restricts their application to a fraction of cells that harbor these marks and to the identification of large cell lineages. These approaches are therefore not suited to analyze small and mid-sized lineages and poorly resolve branching points during divergent cell fate decisions. In particular, identifying close lineage relationships to study cell fate decisions in vivo and in human samples remains challenging.

Here, we quantitatively dissect the maintenance of gene expression in cell lineages for a broad range of cell types. We leverage genes with particularly stable expression over time to develop Gene Expression Memory-based Lineage Inference or GEMLI, a computational tool that allows identifying cell lineages related to several cell divisions based solely on scRNA-seq data.

## Results

### Cells belonging to the same lineage tree are similar in their gene expression profiles

**Fig. 1:**
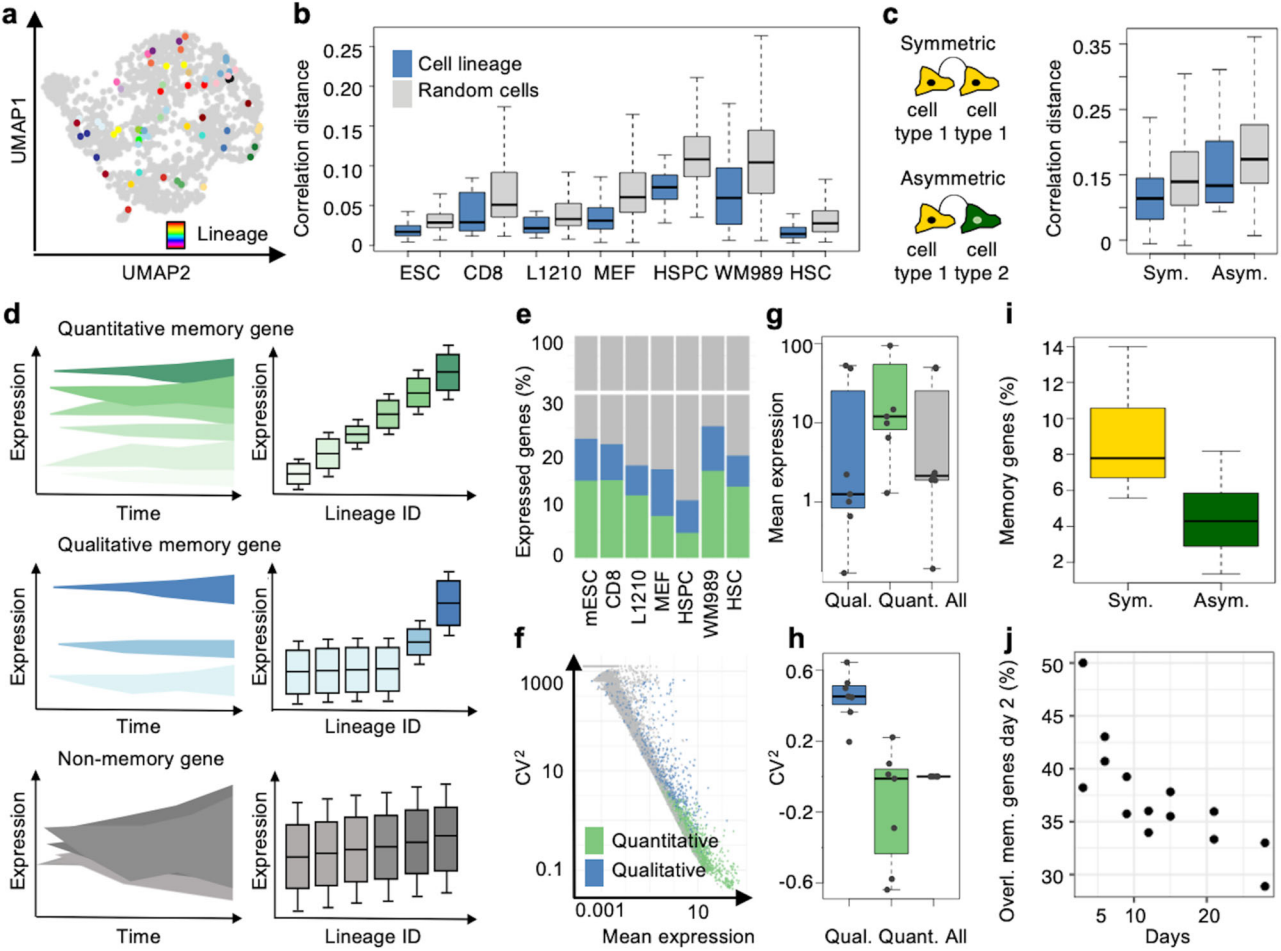
Memory genes drive the transcriptomic similarity of related cells across cell types. **a** UMAP of the lineage-annotated mESC scRNA-seq dataset with 25 random colored lineages. **b** Boxplot of the correlation distance for related cells (blue), and randomly sampled cells (gray; 100 repetitions) in different cell types (*n* = 1 dataset for each cell type). **c** Boxplot as in **b** for lineages encompassing one single cell type (symmetric; sym.) and lineages encompassing several cell types (asymmetric, asym.) for one HSPC dataset. **d** Illustration of the temporal pattern of expression levels and variability of quantitative memory genes, qualitative memory genes, and non-memory genes across cell generations and lineages, respectively. **e** Percentage of expressed genes (gray) that are categorized as quantitative (green) and qualitative (blue) memory genes across cell types (*n* = 1 dataset for each). **f** Relation of CV^2^ and mean gene expression in the mESC dataset. The different memory gene categories are colored as in **e**. **g** Mean gene expression for different memory gene categories and all genes across cell types as in (**b**; *n* = 7 datasets, qual=qualitative, quant=quantitative). **h** Same representation as in **g** for CV^2^ of gene expression. **i** Memory genes as a percentage of expressed genes for lineages being composed of one cell type (symmetric; sym.) and lineages encompassing several cell types (asymmetric; asym) at day 4 of HSPC differentiation (*n* = 22 datasets). **j** The overlap between memory genes of day 2 and day 5–30 over the course of MEF reprogramming. Overlap is the percentage of the memory gene number at day 5–30, respectively (*n* = 2 dataset for each timepoint). Boxes: intervals between the 25th and 75th percentile and median (horizontal line). Error bars: 1.5-fold the interquartile range or the closest data point when no data point is outside this range. Source data are provided as a Source Data file.

We analyzed the transcriptome-wide stability of gene expression over around 1-5 cell divisions using lineage-annotated scRNA-seq datasets of mouse embryonic stem cells (mESCs; Fig. S1a–c), primary mouse embryonic fibroblasts (MEF)^9,33^, primary mouse CD8+T-lymphocytes (CD8)^21^, lymphocytic leukemia cells (L1210)^21^, primary mouse hematopoietic stem and progenitor cells (HSPC; differentiating into >10 mature cell types)^12,33^, mouse hematopoietic stem cells (HSC) ^23^, and human melanoma cells (WM989)^4^, grown for 2–14 days in various culture conditions (Supplementary Data File 1 and Fig. S1d, e for culture times, conditions and dataset sizes, over 120 datasets in total). Two datasets contained specifically sister cells, in all other datasets 2-cell lineages corresponded to random samples from larger lineages (Fig. S1f–g). For all cell types analyzed, we observed a higher correlation (lower correlation distance) of the whole transcriptome for related cells as compared to randomly sampled cells (Fig. 1a, b). Related cells were also more similar in their transcriptomic velocity^34^, complexity (number of expressed genes), and cell cycle phase (Fig. S2a–g). Importantly, for differentiating HSPC, both lineages composed of a single cell type (symmetric) and lineages made of several cell types (asymmetric) were more similar in their transcriptome than random cells (Fig. 1c and Fig. S2h). To further determine the time spans of transcriptomic similarity persistence, we analyzed lineage-annotated data of MEF cultured for up to 30 days. Gene expression correlation of related cells remained high for extended timespans (>20 days) (Fig. S2i), in line with recent reports^7,10,35,36^. In summary, both self-renewing and differentiating cells exhibit substantial gene expression memory over several cell generations.

### Gene expression memory increases gene expression variability in large cell populations

We next asked to which extent transcriptome similarity of small cell lineages impacts gene expression variability in the whole cell population. To do so, we fitted a linear model to compute the total variation for each gene from the variation across pooled lineages. We then used this model to estimate the total variation in a control dataset with permuted lineage labels, and compared the total variation of the real and the control datasets. We found that transcriptomic similarity of cell lineages had a substantial impact on gene expression variability in all cell types, in particular for highly expressed genes (Fig. S3a), with the median standard deviation being on average 4.2% higher (+/– S.D. 3.3) overall transcripts and 19.1% higher (+/– S.D. 15) for highly abundant genes (Fig. S3b). Therefore, while gene expression memory limits gene expression variability within small cell lineages, it is an important source of gene expression variability in large cell populations.

### Thousands of memory genes display stable gene expression levels through cell division

The transcriptomic correlation of closely related cells was driven by ca 20% of expressed genes (on average 3668 +/– S.D. 1131) with particularly stable gene expression levels across cell divisions, which we called memory genes (Fig. 1d, e). We defined memory genes as genes having a significantly higher (*p* < =0.05) variability (coefficient of variation squared (CV^2^)) in their mean expression across cell lineages compared to random cell samples. Memory genes were only partly overlapping but shared characteristic expression distributions across datasets. Some memory genes were widely and highly expressed but showed very conserved expression levels within individual cell lineages (2286 +/– S.D. 890). Other memory genes were highly variable, and only expressed in a small number of cell lineages (1382 +/– S.D. 413). We named these quantitative and qualitative memory genes, respectively (Fig. 1f–h; Figs. S4 and S5 and Supplementary Data file 2). Memory genes, and in particular quantitative memory genes, were enriched for housekeeping functions (Fig. S5 and Supplementary Data file 3). Memory genes were also present for lineages encompassing several cell types (Fig. 1i), highly shared across cell types within a culture condition (Fig. S4f–g), and present over longer culture time spans (>20 days; Fig. 1j and Fig. S4h).

### GEMLI uses shared memory gene characteristics for de novo lineage predictions from scRNA-seq datasets

**Fig. 2:**
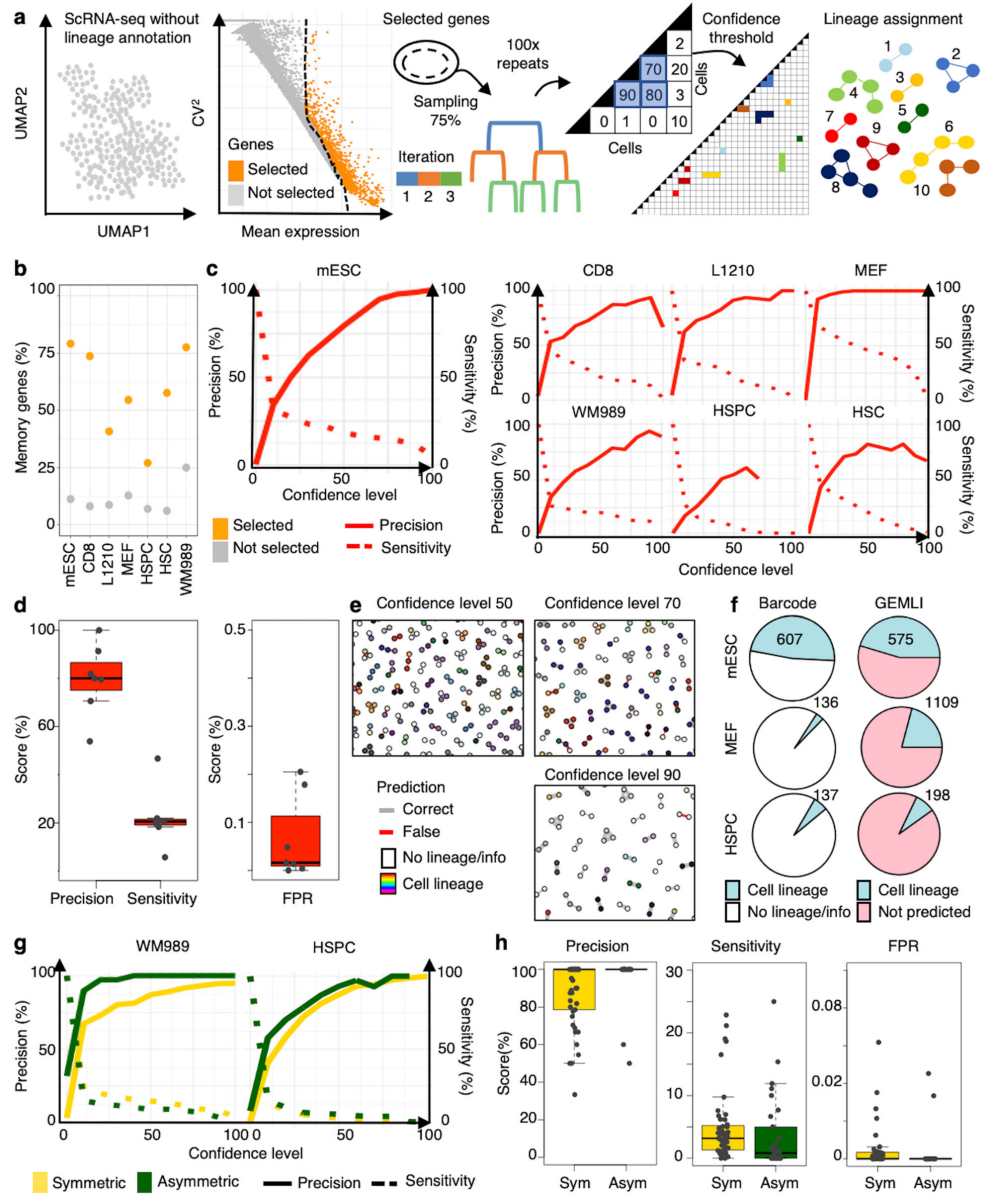
GEMLI predicts cell lineages from gene expression within and across cell types. **a** Schematic of the GEMLI lineage prediction pipeline. **b** Fraction of memory genes selected based on variability and mean expression in the indicated cell types (*n* = 1 dataset for each). **c** Precision-sensitivity curves of lineage predictions in different cell types as in **b**. **d** Precision, sensitivity (left) and FPR (right) of lineage predictions at confidence level 50 for datasets as in (**b**; *n* = 7 datasets). **e** Correct and false (line color) predicted lineages with respect to the barcode lineages (fill color) at different confidence levels in the mESC dataset. A random subset of the lineage predictions is shown. **f** Pie chart of datasets colored according to the presence of barcode- (left) and GEMLI- (right) lineage annotation at a confidence level of 50 (*n* = 1 for each cell type). The number of recovered lineages is indicated. **g** Precision-sensitivity curve for predictions of lineages composed of one cell type (symmetric) or two cell types (asymmetric) in the HSPC (*n* = 44) and WM989 datasets (*n* = 8). Mean is represented. **h** Precision (left), sensitivity (middle), and FPR (right) of lineage predictions at confidence level 50 for symmetric (sym.) and asymmetric (asym.) lineages in the HSPC and WM989 datasets as in **g**. Boxes: intervals between the 25th and 75th percentile and median (horizontal line). Error bars: 1.5-fold the interquartile range or the closest data point when no data point is outside this range. Source data are provided as a Source Data file.

Based on the characteristic expression distributions of memory genes across cell types, we developed GEMLI (for Gene Expression Memory-based Lineage Inference) to predict cell lineages related over several cell divisions de novo in scRNA-seq datasets (Fig. 2a). First, genes with a high expression mean and high variability (mean-corrected CV^2^) are selected. This highly enriches memory genes (Fig. 2b, for comparison to genes used in standard scRNA-seq analysis and optimization of gene selection see Figs. S6 and 7; and Methods). Next, a custom repetitive, iterative hierarchical clustering allows estimating whether cells belong to the same lineage (Fig. 2a; see Table S1 for the main conceptual differences between GEMLI and other clustering algorithms). Briefly, cells are clustered iteratively on random subsets of the selected genes until being assigned to a cluster of predefined size (lineage size parameter; default 2–3 cells). By repeating this clustering, every cell pair is assigned a level of confidence for belonging to the same lineage, based on the number of times it clusters together across individual predictions. Finally, a confidence level threshold is set to define multi-cellular lineages (Fig. 2a). Importantly, lineages are not technically restricted in size and can be larger than the cluster size of 2–3 cells. When applying this approach to different datasets, the precision and sensitivity of small cell lineage predictions reached on average 80% +/– S.D. 15%, and 22% +/– S.D. 12%, respectively, at a confidence level of 50. The false positive rate (FPR) of predictions stayed consistently under 1% with an average of 0.07% +/– S.D. 0.08% at a confidence level of 50 (Fig. 2c–e and Fig. S8). GEMLI performed consistently better than other gene selection and lineage assignment strategies, including a k-nearest neighbor (kNN) clustering and the training of a neural network (Fig. S7c, g and Fig. S9). Crucially, as GEMLI allows to predict cell lineages for cells in which cellular barcoding fails, the number of lineage-annotated cells was either comparable or substantially increased as compared to barcode-based approaches (Fig. 2f). Changing prediction parameters, such as the fraction of genes sampled for each clustering (Fig. S10), can further optimize precision at the expense of sensitivity or vice versa, depending on which measure is deemed more important for the downstream analysis. GEMLI allows the identification of both symmetric and asymmetric lineages, i.e., lineages in which cells belong to one or different cell types (Fig. 2g, h and Fig. S11) and lineages of different sizes (Figs. S12 and S13). Furthermore, lineage predictions were also possible over extended time spans at high precision but with a decreasing recovery, in line with a gradual decline of gene expression memory over time (Figs. S14 and S4h; and Fig. 1j). GEMLI performed well for datasets within the recommended size and sequencing depth ranges. Notably, GEMLI performed well in datasets with a sequencing depth >5000 reads/cell (all but the HSPC datasets; Fig. S15)^37–39^. Altogether, GEMLI allows for accurate prediction of cell lineages related over several cell divisions solely from scRNA-seq datasets.

### GEMLI identifies memory gene categories, cell fate decisions, and associated gene expression programs

**Fig. 3:**
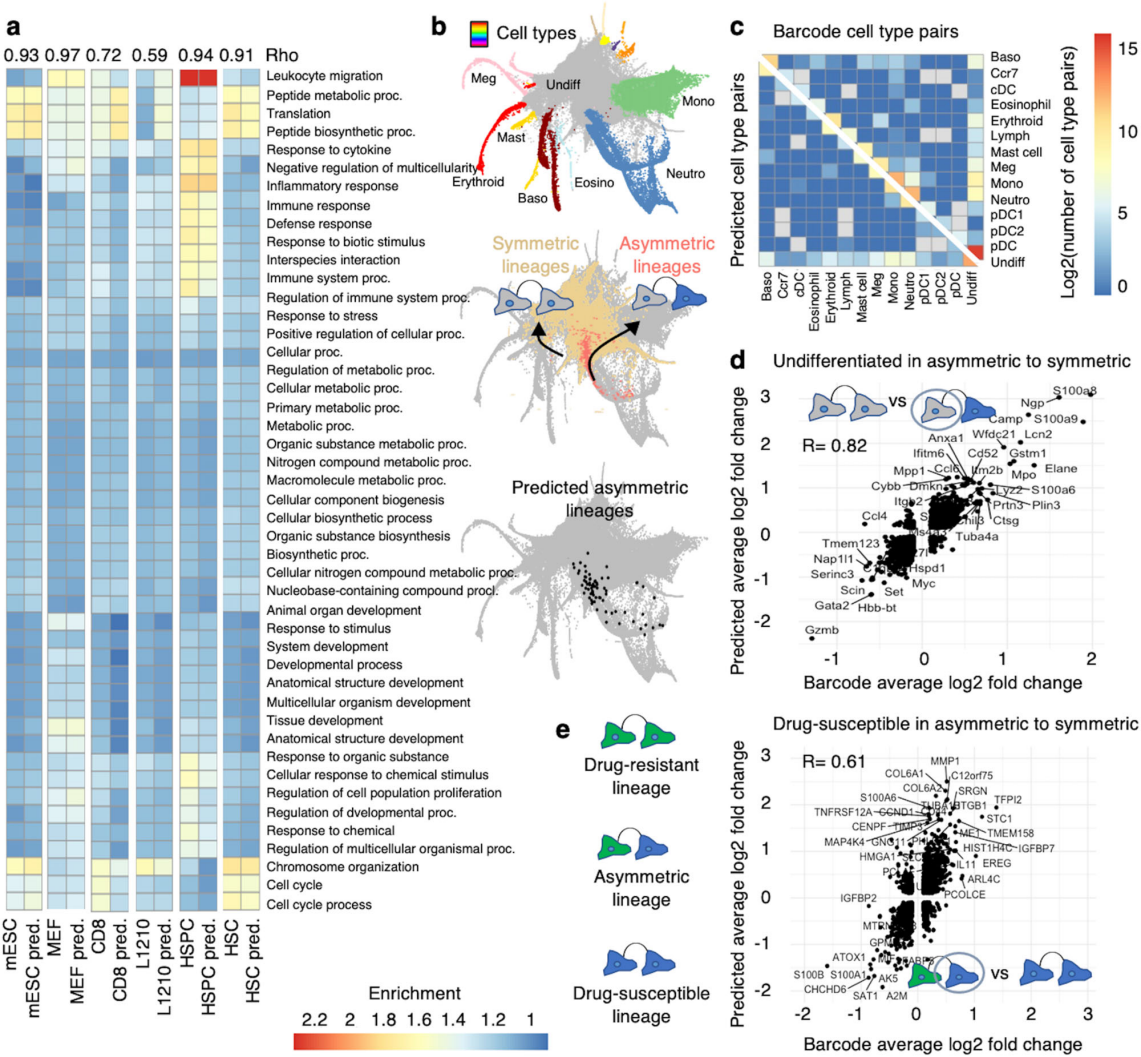
GEMLI lineage predictions allow retrieving memory GO-terms and quantifying diverging cell-fate decisions. **a** Top 10 enriched GO-terms in memory genes called on barcode or predicted lineages (pred.) at confidence level 30 in different cell types (*n* = 1 dataset each). All terms that are among the top 10 in any cell type are shown. Spearman rank correlation is indicated. Proc.=process. **b** SPRING graph of HSPC datasets (*n* = 44) annotated by cell types (top), highlighting ground truth undifferentiated symmetric (beige) and undifferentiated-neutrophil asymmetric (pink) lineages (middle), or highlighting undifferentiated-neutrophil asymmetric lineage predictions at confidence level 70 (bottom). **c** The number (sum) of cell pairs in the barcode (top right) and predicted (bottom left) cell lineages in all cell type combinations in the HSPC datasets as in **b**. **d** Average log2 fold-change for DEG called between barcode and predicted undifferentiated cells in symmetric and asymmetric cell lineages in one day 4 HSPC dataset. Dots represent DEGs. Spearman rank correlation is given. The 20 highest and 10 lowest enriched genes are named. Gray: undifferentiated, blue: neutrophil. **e** Average log2 fold-change for DEG called between barcode and predicted drug-susceptible cells in symmetric and asymmetric cell lineages across WM989 datasets (*n* = 8). Green: drug-resistant, blue: drug-susceptible. A confidence level of predictions in **c**–**e** is 50. For the full spelling of abbreviated cell type names mentioned in panels **b**, **c**, see Methods. Source data are provided as a Source Data file.

We then asked how reliably GEMLI identifies categories of genes displaying gene expression memory in small lineages. To do so, we compared memory genes and associated GO-terms from GEMLI predictions and ground truth lineage annotation and found them to be highly correlated for all datasets (Fig. 3a; Fig. S16a–c and Supplementary Data file 5). Next, we tested the ability of GEMLI to identify symmetric vs. asymmetric cell fate decisions. In both WM989 melanoma cell and HSPC datasets (total of 52 datasets, low sequencing depth for HSPCs), the fraction of related cells among different symmetric and asymmetric cell pairs correlated well for GEMLI and barcode lineages (Fig. 3b, c and Fig. S16d–j). The prevalence of related cells among drug-susceptible and -resistant melanoma cells, as well as 10 possible hematopoietic cell types could be distinguished. Also, differentially expressed genes (DEG) between asymmetric and symmetric cell type pairs were recovered well by analyzing GEMLI lineages (Fig. 3d, e and Fig. S17). In the HSPC data, DEG called between undifferentiated cells in symmetric undifferentiated and asymmetric undifferentiated-neutrophil cell pairs in the ground truth and predictions correlated highly (Fig. 3d). In the WM989 cells, DEG analysis of GEMLI lineages identified genes specifically expressed in drug-susceptible cells being part of lineages with drug-susceptible and drug-resistant members, i.e., lineages in which cells recently switched to a resistant phenotype (Fig. 3e). While kNN performed similarly to GEMLI in identifying the prevalence of cell pairs, it performed less so for the recovery of correct DEGs (Figs. S16i, j and  S17a–d). For both analyses, GEMLI performed much better than random cell pair selections (Figs. S16g, h and S17a–f). In line with recent reports^4,12,23,40,41^, a trajectory analysis did not recover asymmetric lineage-associated DEG (Fig. S17g–j). This highlights the utility of GEMLI for downstream analyses that are commonly restricted to barcoded datasets, even in the case of datasets with a low sequencing depth.

### GEMLI predicts lineages in lung metastases of pancreatic cancer

**Fig. 4:**
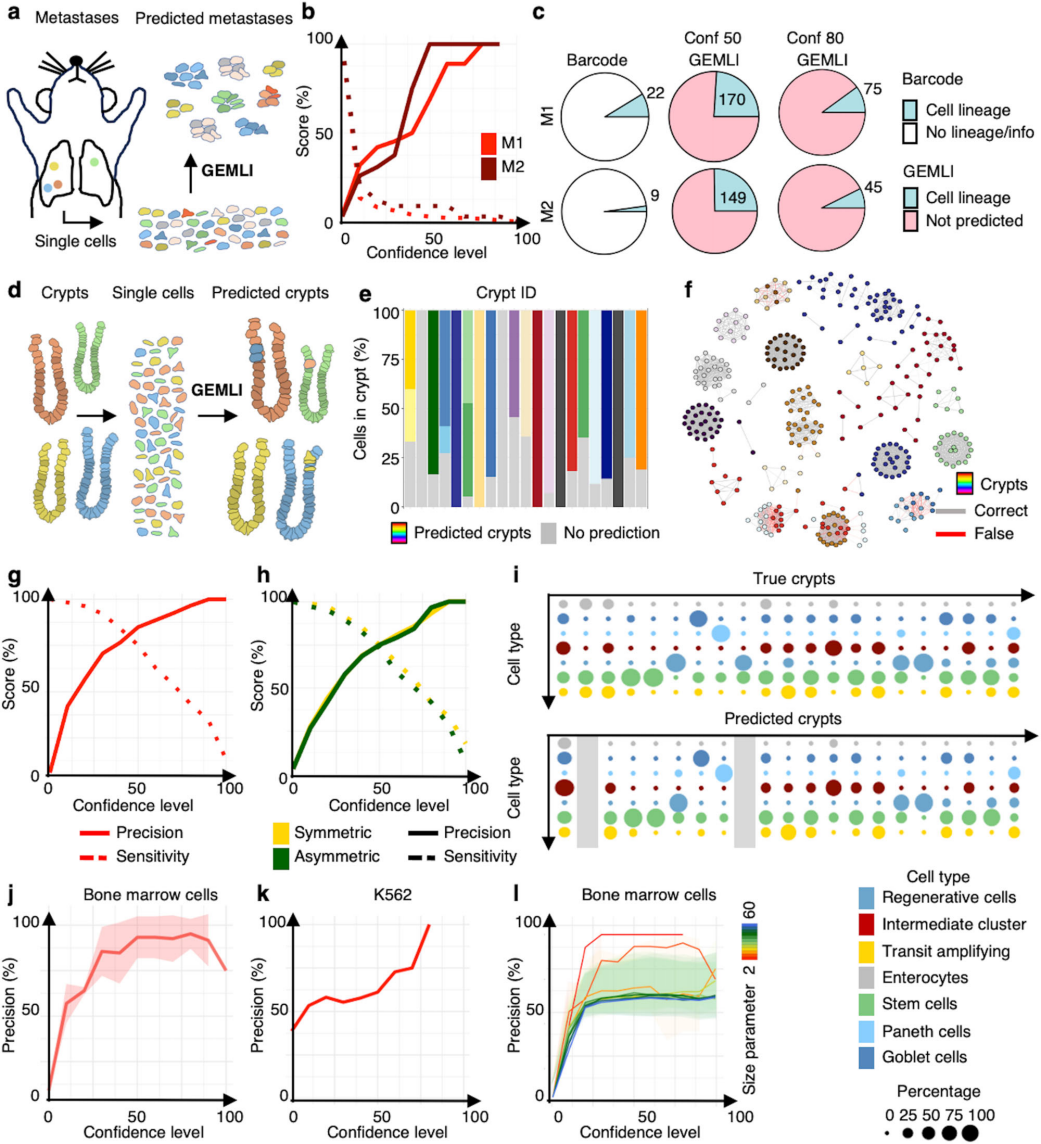
GEMLI correctly assigns cells to individual metastases, crypts, and mitochondrial variant-defined lineages in vivo. **a** Scheme of GEMLI lung metastasis predictions. **b** Precision (line) and sensitivity (dotted line) as a function of confidence level for GEMLI predictions in lung metastases of two mice tested against the CRISPR barcode lineage information. Line: mean; Shade: S.D. **c** Pie chart of the lung metastases data of two mice (M1, M2) as in **b** colored according to the presence of CRISPR barcode- (left) and GEMLI- lineage annotation at a confidence level of 50 (middle) and 80 (right). The number of recovered lineages is indicated. Conf=confidence level. **d** Schematic of GEMLI crypt predictions. **e** Percentage of cells predicted as individual lineages for each crypt in the crypt dataset (*n* = 1) at confidence level 70. **f** Predictions of crypts at confidence level 70. **g** Precision-sensitivity curve for predictions in the crypt dataset. **h** Precision-sensitivity curve for predictions of cell pairs composed of one cell type (symmetric) or two cell types (asymmetric) in the crypt dataset. **i** The percentage of cells (size) belonging to the indicated cell type (color) for individual crypts (top) and for predicted lineages at confidence level 50 (bottom) aligned to their crypt of origin. **j** Precision-sensitivity curves of lineage predictions in bone marrow cells using mitochondrial variants as ground truth lineages (*n* = 3 datasets). Line: mean; Shade: S.D. **k** Precision-sensitivity curves of lineage predictions in K562 cells (*n* = 1 dataset) as in **j**. **l** Precision-sensitivity curves of lineage predictions in bone marrow cells using mitochondrial variants as ground truth lineages as in **j** with lineage size parameters up to the maximal ground truth lineage size. Line: mean; Shades: S.D. All crypt predictions are of 5-40 cells and were run with a lineage size parameter of 2–40. Source data are provided as a Source Data file.

Next, we tested whether GEMLI could identify small and mid-sized lineages in vivo. We used data from mouse pancreatic cancer lung metastases, which have been traced through evolvable CRISPR barcoding over a short timespan^42^. As barcodes here had only four weeks to evolve, CRISPR editing frequency of barcodes is high and the lung metastases develop late, this ground-truth data is suitable for comparison with GEMLI predictions. At a confidence level of 80, GEMLI reached 100% precision while assigning more cells to lineages than the barcoding data (Fig. 4a–c).

### GEMLI assigns cells to individual in vivo-derived crypts and organoids

Further, we tested GEMLIs ability to reconstruct in vivo and in vitro-derived multicellular structures that originate from a single or few stem cells using scRNA-seq data of intestinal organoids and crypts, for which the individual structure of origin (crypt or organoid) is known for each cell (ref. ^43^, Supplementary Data file 1). We ran GEMLI on the pooled scRNA-seq datasets and analyzed whether inferred lineages fall within individual crypts and organoids. GEMLI resulted in high percentages of cells correctly assigned to their crypt or organoid of origin with default lineage size parameters and parameters in the ground truth lineage size range, allowing the recovery of mid-sized lineages (Fig. 4d–g; Figs. S12a, d and S18 for organoids). As ground truth lineage sizes might be unknown when applying GEMLI, we tested the stability of predictions for different lineage size parameters. Taking the maximal ground truth lineage size as the upper lineage size parameter value coincided with a maximal cluster stability of predictions, allowing us to estimate the size distribution of crypts and organoids de novo (Fig. S19). GEMLI predictions allowed highly reliable inference of the cell type composition of individual structures by identifying both symmetric and asymmetric cell pairs (Fig. 4h, i and Fig. S20). This demonstrates that GEMLI can recover the cellular composition of individuals in vivo and in vitro segregated mid-sized multicellular structures from pooled datasets.

### GEMLI can be combined with other lineage-tracing approaches

We next combined GEMLI with lineage inference based on mitochondrial variants to study lineages at broader time scales. Mitochondrial variants allow identifying cell lineages in 3’ scRNA-seq datasets after enrichment of mitochondrial transcripts^27^. The scarce occurrence of mitochondrial mutations however restricts this approach to the assignment of cells to large lineages, i.e., cells related over long time spans (weeks to months). We tested if GEMLI could refine lineage trees inferred from mitochondrial variants on datasets of primary human bone marrow cells and K562 myelogenous leukemia cells^27^. Importantly, in these experiments only a small sample from a large population was sequenced, resulting in the loss of a large fraction of cells from small lineages and thus inherently limiting their identification. Nevertheless, GEMLI recovered small cell lineages in all datasets and these fell with high precision within the large mitochondrial-variant inferred lineages (Fig. 4j, k). The precision stayed also high for predictions up to the maximal size of the mitochondrial-variant inferred lineages, confirming GEMLIs utility in refining lineage trees obtained with other techniques (Fig. 4l).

### GEMLI characterizes gene expression memory in human breast cancer

**Fig. 5:**
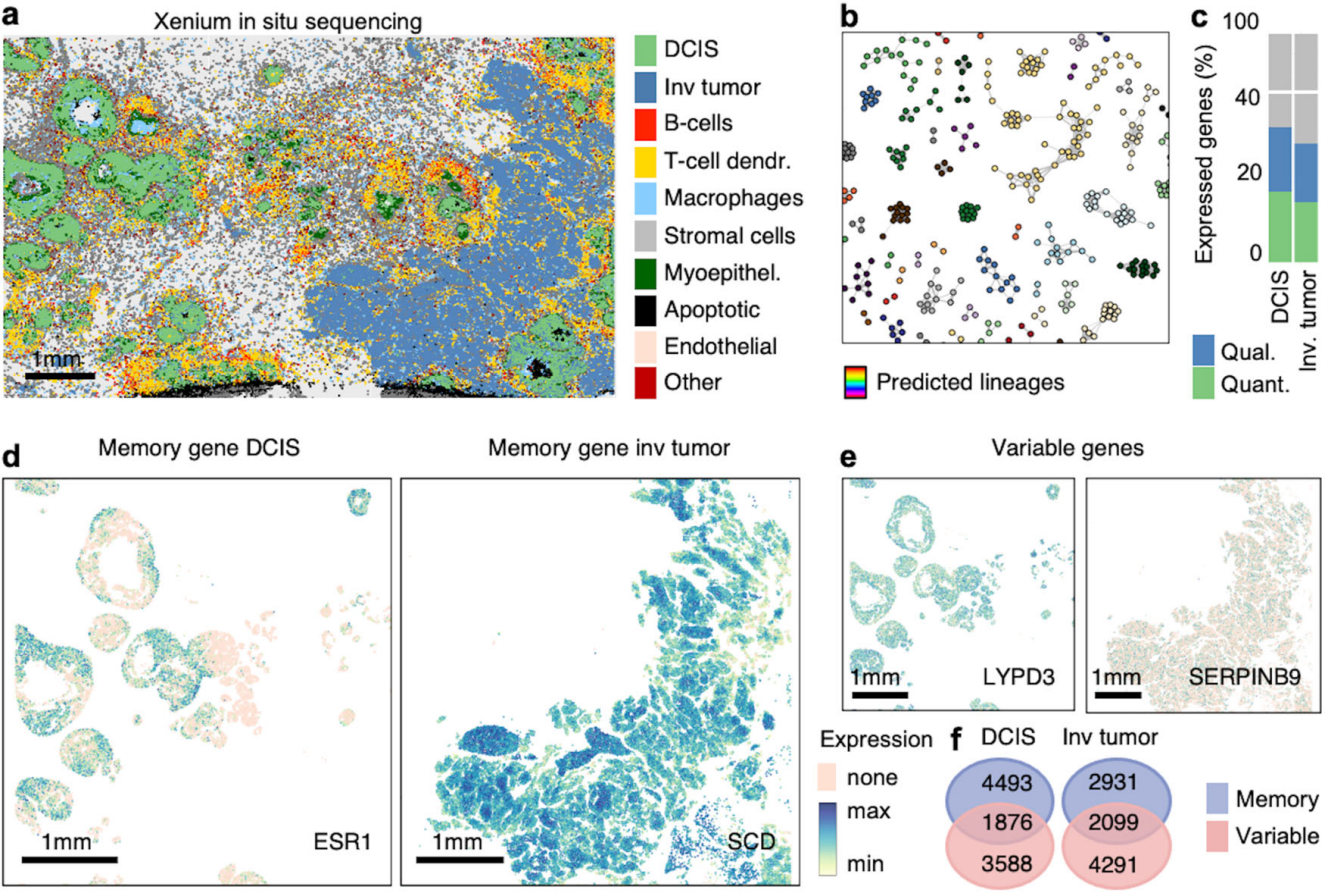
GEMLI predictions identify lineages in breast cancer nodules. **a** Breast cancer Xenium in situ sequencing map colored by cell type. **b** A random subset of predicted lineages within the DCIS cells of the breast cancer scRNA-seq dataset at confidence level 50. **c** Percentage of expressed genes categorized as quantitative (quant.) and qualitative (qual.) memory genes in the breast cancer scRNA-seq dataset as in **b**. **d** In situ map of breast cancer Xenium data for ESR1 and SCD expression which are memory genes in DCIS and invasive tumor lineages respectively. **e** Maps as in **d** for two variable genes. **f** Overlap of memory and variable genes in the breast cancer scRNA-seq dataset. All predictions in b-e are with lineage size parameters of 2–20 cells. The invasive tumor is abbreviated as the inv tumor in all panels. In **a** T-cell dendr. = T-cell dendritic cell and Myoepithel. = Myoepithelial cell. Source data are provided as a Source Data file.

Next, we applied GEMLI to a primary human breast cancer tissue sample dataset^44^, in which other lineage tracing approaches (such as cellular barcoding) cannot be applied or would only call large lineages (mitochondrial-variant-based lineage inference). The dataset was generated from formalin-fixed, paraffin-embedded breast cancer tissue and encompasses scRNA-seq (31,364 expressed genes) and Xenium in situ sequencing data (313 genes) of serial sections^44^. We assigned 10 cell types identified in the Xenium data to the scRNA-seq data using supervised machine learning (Fig. 5a), and applied GEMLI to ductal carcinoma in situ (DCIS) and invasive tumor cells separately (Fig. 5b). Prediction stability for DCIS cells suggested lineage sizes up to 50 cells, in line with their nodule character and size in the spatial data, and resembled the multicellular crypt or organoid profiles (Fig. 5a and Fig. S21, size considering 2,3% recovery of DCIS cells in scRNA-seq). In contrast, cluster stability of predicted invasive tumor cell lineages was more similar to cultured cells without a clear spatial organization (Fig. S21a). Memory genes called on predicted DCIS and invasive tumor cell lineages (Fig. 5c) were characterized by distinct GO terms, such as response to estrogen or cell-substrate adhesion, respectively (Fig. S22a). Memory genes for which Xenium in situ data was available showed visible patches of similar gene expression, in line with lineage-specific expression (Fig. 5d). Crucially, the spatial distribution of highly variable, non-memory genes was characterized by a “salt and pepper” aspect (Fig. 5e), in line with both gene groups only partly overlapping (Fig. 5f).

### GEMLI allows identifying early stages of progression to invasive cancer in vivo

**Fig. 6:**
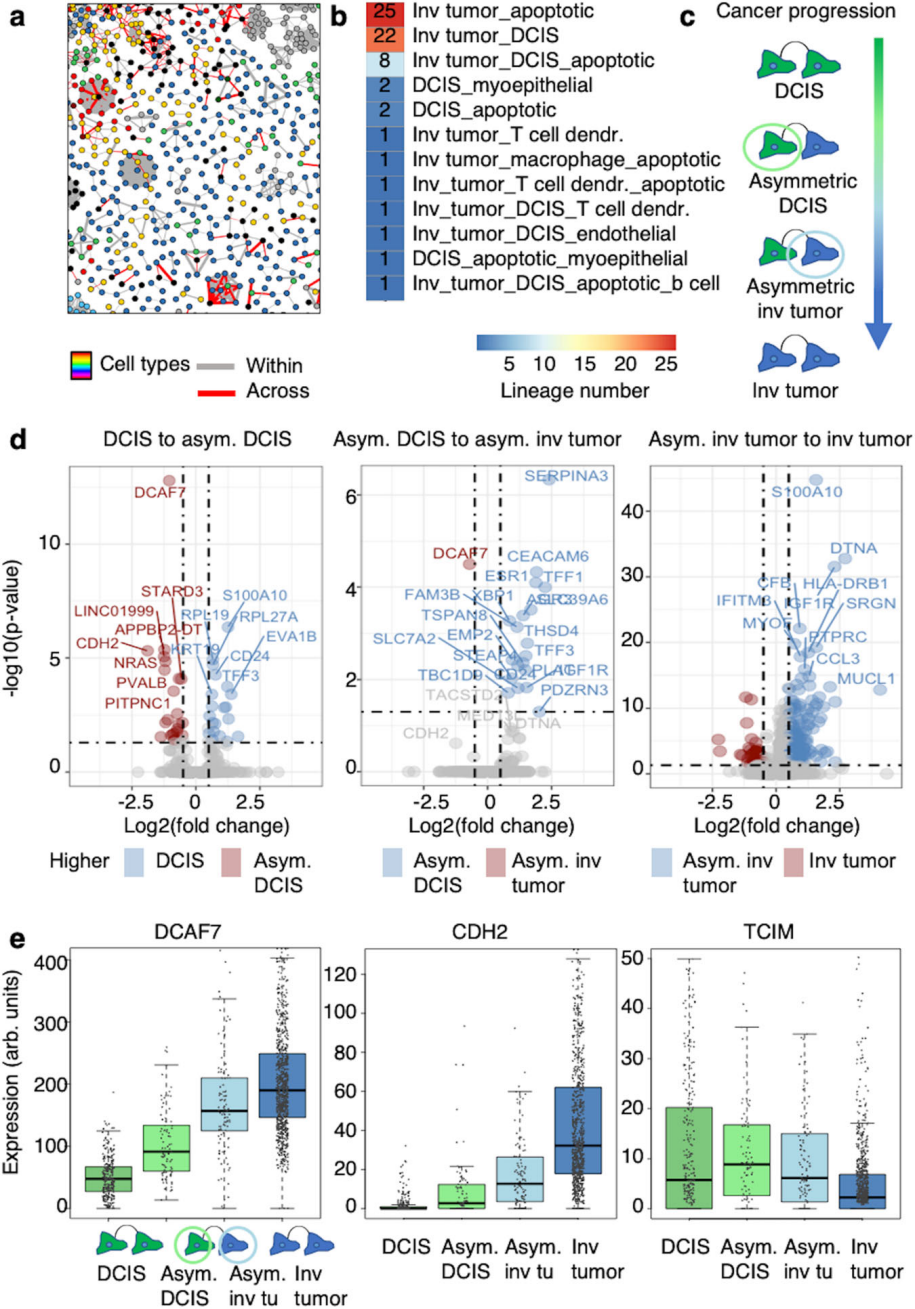
GEMLI identifies breast cancer cells in lineages switching to an invasive phenotype. **a** A subset of predicted lineages with respect to cell type annotation at confidence level 50 in the breast cancer scRNA-seq dataset. Gray and red lines indicate lineage relationships within one or across several cell types, respectively. **b** Number of DCIS and invasive tumor lineages with members belonging to other cell types for lineage predictions as in **a**. **c** Scheme of lineage cell type identities occurring during breast cancer progression towards invasiveness (green: DCIS, blue: invasive tumor). **d** Volcano plots for DEG called between DCIS and invasive tumor cells predicted to be part of symmetric and asymmetric (asym.) cell lineages in the breast cancer scRNA-seq dataset as indicated. The top 9 highest and lowest enriched genes are named. Predictions at confidence level 50. The *p*-value indicated was generated by Seurats FindMarker function based on a Wilcoxon Rank Sum Test. **e** Expression of selected DEG as in **d** across DCIS and invasive tumor cells part of symmetric and asymmetric (asym.) lineages as indicated. All predictions are with lineage size parameters of 2–20 cells. Invasive tumor is abbreviated as ‘inv tumor’ or ‘inv tu’ in all panels. Arb. units = arbitrary units. Boxes: intervals between the 25th and 75th percentile and median (horizontal line). Error bars: 1.5-fold the interquartile range or the closest data point when no data point is outside this range. Source data are provided as a Source Data file.

Finally, we applied GEMLI to predict lineages on the whole breast cancer scRNA-seq dataset to study progression towards invasiveness. While most GEMLI lineages were confined within one cell type or shared with apoptotic cells, GEMLI also predicted lineages with both DCIS and invasive tumor cell members (Fig. 6a, b and Fig. S22b, c, around 10% of cancer cell lineages). We hypothesized that these asymmetric lineages contained cells that recently switched to an invasive phenotype. We analyzed genes characterizing these lineages by calling DEG on DCIS cells within asymmetric and symmetric lineages, as well as DEG between lineage types of later stages in cancer progression (Fig. 6c, d and Fig. S22d). Genes enriched in DCIS cells from asymmetric lineages included known markers of cancer invasiveness such as *CDH2*^45^, but also genes with unknown roles in cancer progression, such as *DCAF7* and *LINC01999* (Fig. 6d). While, e.g., *DCAF7* and *CDH2* displayed continuous changes across lineage types occurring during cancer progression, others such as *TCIM* or *BRIP1* were expressed transiently at higher levels in asymmetric or only in symmetric (i.e., only invasive tumor cell) lineages, respectively (Fig. 6e and Fig. S22e). This demonstrates the unique ability of GEMLI in dissecting lineage identities and gene expression changes in specific lineage types occurring during the progression of human tumors towards invasiveness.

## Discussion

GEMLI allows predicting small to medium-sized cell lineages related over several generations solely from scRNA-seq datasets, based on genes that maintain their expression level through cell divisions. This is a shift away from experimental lineage tracing techniques based on genetic marks or physical cell lineage separation and extends lineage annotations of small and medium-sized lineages to in vivo settings.

GEMLI is applicable to any scRNA-seq dataset and requires only exonic reads. This alleviates the need for experimental lineage assignment procedures implemented prior to sequencing and thereby expands lineage annotation to contexts where cellular barcoding or other lineage assignment approaches are challenging or prohibitive, such as primary cells or human biopsies. It also avoids cellular stress caused by cell picking or genetic engineering required for manual and cellular-barcoding-based lineage tracing, respectively. GEMLI is particularly promising for its ability to identify small lineages in which cells are related over about 1-5 cell divisions. However, GEMLI will generally perform less robustly in cell populations that contain only distantly related cells. Some systems might allow for a longer gene-expression memory-based lineage tracing^9,10,36^. While GEMLI performed well on intestinal organoids that are highly variable in size, we cannot exclude that large variability in growth rates between lineages may decrease its performance.

We show that in line with individual reports^7,10,35,36^, gene expression memory is present in various cell types and populations in both self-renewal and differentiation conditions. GEMLI is therefore applicable to all cells of a scRNA-seq dataset, in contrast to cellular barcoding-based approaches which are limited to cells that (i) express the barcode and (ii) for which the barcode is recovered. GEMLI thus allows to recovery of a comparable or higher number of lineages than in public cellular barcoding scRNA-seq datasets. GEMLI also compares favorably to cell-picking approaches, which are difficult to scale up and therefore mostly encompass small cell numbers^21,23,25^.

Lineage inference approaches based on naturally occurring genetic marks can identify lineages over time spans of weeks or months but require high coverage and sequencing depths or the enrichment of specific transcripts^26–32^. Trajectory inference algorithms can predict gene expression changes on the order of hours but do not predict lineages^34,41,46,47^. GEMLI thus fills a methodological gap and shall become a powerful tool to refine lineage trees in combination with other lineage tracing approaches.

With its resolution at the scale of several cell divisions, GEMLI is ideally suited to assess cell fate decisions during differentiation, response to stimulus, homeostasis, and regeneration, and allows correctly assigning a broad diversity of cell types to individual complex structures such as intestinal crypts and organoids. In these cases, the physical proximity of lineage-related cells allows the sharing of a similar microenvironment, which may further increase GEMLIs performance. GEMLI is therefore promising to reconstruct other structures emerging from a common ancestor or a small pool of stem cells, such as glands or spatially restricted areas of the skin. In the context of human breast cancer, we illustrate GEMLIs applications by identifying DCIS nodules, as well as determining previously unknown gene expression changes in lineages encompassing cells at the transition towards an invasive phenotype. Altogether, GEMLI opens new perspectives in interrogating the impact of close lineage relationships in a broad range of biological contexts in vivo.

## Methods

### Cell culture

CGR8 ES cells (Sigma, Cat307032901-1VL) were routinely cultured at 37 °C and 5% CO_2_ on 10 cm dishes coated with 0.1% gelatin type B (Sigma, Cat#G9391-100G) in 10 ml GMEM (Sigma, Cat#G5154‐500 ML) supplemented with 10% ES cell‐qualified fetal bovine serum (Gibco, Cat#16141‐079), 1% nonessential amino acids (Gibco, Cat#11140‐050), 2 mM l‐glutamine (Gibco, Cat#25030‐024) and sodium pyruvate (Sigma, Cat#S8636‐100 ML), 100 μM 2‐mercaptoethanol (Sigma, Cat#63689‐25 ML‐F), 1% penicillin and streptomycin (Pen/Strep) (BioConcept, Cat#4‐01F00‐H), homemade leukemia inhibitory factor (LIF), CHIR99021 (Merck, Cat#361559‐5MG) at 3 μM and PD184352 (Sigma, Cat#PZ0181‐25MG) at 0.8 μM. Cells were passaged by trypsinization (2 ml of trypsin, Sigma, Cat#T4049‐100 ML) every 2–3 days at a ratio of 1:10. For scRNA-seq experiments, cells were switched to N2B27 + 2i/LIF medium 2 passages beforehand. N2B27 + 2i/LIF medium was composed of a 1:1 mix of DMEM/F12 (Gibco, Cat#11320‐033) and Neurobasal medium (Gibco, Cat#21103‐049), supplemented with N2 (Gibco, Cat#17502‐001), B27 (Gibco, Cat#17504‐001), 1% Pen/Strep (BioConcept, Cat#4‐01F00‐H), 2 mM L-glutamine (Gibco, Cat#25030‐024), 100 μM 2-mercaptoethanol (Sigma, Cat#63689‐25 ML‐F), LIF, CHIR99021 (Merck, Cat#361559‐5MG) at 3 μM and PD184352 (Sigma, Cat# PZ0181‐25MG) at 0.8 μM. Cells were split every 2–3 days at a ratio of 1:10, using 2 ml of accutase (Innovative Cell Technologies, Cat#31195) and centrifugation. HEK293T cells were routinely cultured at 37 °C and 5% CO_2_ on dishes in DMEM high glucose medium (Gibco, Cat#41966) supplemented with 10% Fetal bovine serum (Life Technologies, Cat#10270-106) and 1% Pen/Strep (BioConcept, Cat#4‐01F00‐H).

### Lentiviral barcoding library production

The LARRY lentiviral barcoding library^12^ was purchased from Addgene (https://www.addgene.org/pooled-library/camargo-plarry-egfp-barcoding-v1/). The barcodes of the library are composed of 40 base pairs (bp) with 28 random bp separated by 6 fixed bp doublets and are located in the 3’ untranslated region of *EGFP* expressed from the *EF-1ɑ* promoter. The library was amplified and the lentiviral vector was produced according to the associated protocol with small modifications. Briefly, plasmids were introduced into ElectroMAX Stbl4 Competent Cells (Life technologies, Cat#11635018) using MicroPulser Electroporator (Bio-Rad, Cat#1652100) EcoRI program. Cells were incubated for 1 h at 37 °C and spread over 20 large (24.5 × 14.5 cm) Agar+Ampicillin plates (Ampicillin at 100 ug/ml, AppliChem, Cat# A08390025). After 24 h, colonies were harvested through scraping using pre-warmed LB medium supplemented with 100 ug/ml Ampicillin. The resulting 1.5 L bacterial culture was incubated at 37 °C for 2 h and a Maxiprep was performed using a Qiagen MaxiPlus kit (Qiagen, Cat#12963) to a resulting 1 mg of plasmid DNA. A lentiviral vector was produced by transforming the produced LARRY plasmid into HEK293T cells using the Trans-IT 293 transfection reagent (Mirus, Cat#MIR2700). For each of eighteen 10 cm dishes of HEK293T cells, 1.5 ml Opti-MEM (Gibco, Cat#51935), 16 μg plasmid (8 μg LARRY plasmid, 6 μg psPAX2 (Addgene, PRID: Addgene_12260), 2 μg pMD2.G (Addgene, PRID: Addgene_12259)) were mixed with 45 μl TransIT 293 transfection reagent, incubated for 15-30 min at RT, and added to the cells dropwise. Lentiviral vector particles were harvested 48 h and 72 h after transfection. Medium was collected and filtered through a 0.45 μm PVDF filter (Millipore, Cat#SLHV033RS), and centrifuged in a Beckman Optima XL-80K Ultracentrifuge (Beckman, Cat#8043-30-1211) at 140,000 × *g* for 1h 30 min at 4 °C. The supernatant was removed, and pellets were resuspended with 100 μl of GMEM with serum and all additives as above but without 2i/LIF. The lentiviral vector preparation was incubated on ice for 30 min, aliquoted, and stored at –80 °C. Titration of the lentiviral barcoding library was performed on CGR8 cells cultured in GMEM+2i/LIF, with a read-out 5 days after infection.

### Barcode reference library generation

A reference library was made through the sequencing of PCR-amplified barcodes from the LARRY plasmid library in triplicates. 500 ng of LARRY plasmids were taken as input for a two-step PCR using Phusion high-fidelity DNA polymerase (Thermo Fisher, Cat#F-5305) adapted for LARRY from ref. ^48^. The first step amplifies the barcodes and adds Illumina Read1 and Read2 sequences (5’ACACTCTTTCCCTACACG ACGCTCTTCCGATCTTGTGACGTCACAGGTCGACACCAGTCTCATT3′ and 5’GTGACTGGAGTTCAG ACGTGTGCTCTTCCGATCGAGTAACCGTTGCTAGGAGAGACCATA3′). The second step adds the P5 and P7 flow cell attachment sequences and a sample index of 7 bp (P5 5’AATGATA CGGCGACCACCGAGATCTACACTCTTTCCCTACACGACGCTCTTCCGATCT3′ and P7 5’CAAGCAGA

AGACGGCATACGAGANNNNNNNGTGACTGGAGTTCAGACGTGCTCTTCCGATC3′). 200 ng of PCR1 product was taken as input for PCR2. (PCR programs: 98 °C 2 min, 15 cycles of 98 °C 10 s, 67.2 °C (PCR1), 72 °C (PCR2) 30 s, 72 °C 30 s, followed by final elongation 72 °C 5 min and 4 °C indefinitely). In between PCR1 and PCR2 and after PCR2, PCR purification was performed using the QIAquick PCR purification kit (Qiagen, Cat#28106). The mix of the three samples was sequenced on a MiSeq instrument (Illumina) at the Gene Expression Core Facility of EPFL. Sequencing results were first filtered for a perfect match to the plate index pattern using XCALIBR (https://github.com/NKI-GCF/xcalibr). The resulting read files were filtered for a perfect match to the barcode pattern using customized R scripts. The resulting list and the LARRY “barcode_list” available on Addgene (https://www.addgene.org/pooled-library/camargo-plarry-egfp-barcoding-v1/) were merged and used as a reference list.

### mESCs scRNA-seq memory experiment run

CGR8 cells were transduced with 3 ul of the lentiviral LARRY barcoding library per 10 cm dish with 10 ml medium to obtain roughly 1% of GFP^+^ cells. 72 h after infection, live GFP^+^ cells (Fig. S1a; live stain 1:500 propidium iodide solution (BioLegend, Cat#421301)) were sorted on an FACSAria III (BD) at the Flow Cytometry Core Facility of EPFL into Cellcarrier 96-Ultra 96-well plates (PerkinElmer, Cat#6055308). Plates were coated with recombinant human E-cadherin-Fc chimera (BioLegend, Cat#BLG-779904-25ug) to reduce colony formation and thereby limit the potential impact of paracrine and direct cell signaling in regulating gene expression of related cells^49^. Coating was performed at 10 μg/ml for 1 h at 37 °C. After washing the plates with PBS, 1,200 cells were sorted into two wells and both were collected after 48 h for sequencing. Before collection, cells were imaged on an IN Cell analyzer 2200 (GE Healthcare) at the Biomolecular Screening facility of EPFL. ScRNA-seq library preparation was performed on a 10X Genomics Chromium platform of the Gene Expression Core Facility of EPFL using the SingleCell 3’ Reagent Kit v3.1. The sample was sequenced on a Hiseq4000 instrument (Illumina). The experiment was performed once.

### mESC scRNA-seq data processing and cell lineage inference

ScRNA-seq data was analyzed using 10X Genomics Cell Ranger (v. 5.0.1), Seurat (v. 4.1.0), customized R (v. 4.0.2), and Python (v. 3.9.7) scripts. Raw sequencing reads were processed using 10X Genomics Cell Ranger (v. 5.0.1) using default parameters and refdata-gex-mm10-2020-A as reference genome, with or without the *include-introns* option. Cell Ranger outputs a unique molecular identifier (UMI) corrected read count matrix. Cells with a percentage of mitochondrial reads between 1.75 and 7.5 and with more than 10,000 reads were further analyzed. Data was normalized to 40,000 reads per cell (similar to RPM, one normalized count equals one read on median). Lineage barcodes were extracted from the data using the CellTag pipeline^9^ available for download at (https://github.com/morris-lab/BiddyetalWorkflow) and adapted to the LARRY barcode design. Briefly, the CellTag pipeline extracts reads containing a CellTag motif from the processed, filtered, and unmapped reads BAM files produced in intermediate steps of the 10X Genomics Cell Ranger pipeline. To extract LARRY barcodes, the CellTag motif was changed to “([ACTG]{4}TG[ACTG]{4}CA[ACTG]{4}AC[ACTG]{4}GA[ACTG]{4}GT [ACTG]{4}

AG[ACTG] {4})” in all scripts. Barcodes with reads from only one UMI, and without perfect match to the reference library were filtered out. A Jaccard similarity score of >0.7 was used to identify cell lineages. No filtering on the number of barcodes expressed per cell was performed. Lineages were called on unfiltered data. For the mESC dataset, 6 lineages had sizes above 5 cells, which is larger than expected based on cell cluster sizes after 48 h of culture and the expected loss of cell lineage members in the preparation for scRNA-seq. They were therefore excluded from further analysis.

### Processing of public scRNA-seq data

Data from Biddy et al.^9^ was extracted as a BAM file from SRA links specified under GSE99915. BAM files were converted back to fastq format using 10X Genomics’ bamtofastq (v. 1.3.2) and Cell Ranger (v. 5.0.1) was run on the resulting data as described above with or without the *include-introns* option. Cells were filtered on the percentage of mitochondrial reads and the number of reads as indicated in Table S2. Data was normalized to 40,000 reads per cell. Cell lineages were assigned using the CellTag pipeline as specified above with the original CellTag motif of “(GGT([ACTG]{8})GAATTC)”(V1), “(GTGATG([ACTG]{8})GAATTC)”(V2) or “(TGTACG([ACTG]{8})GAATTC)”(V3) and the respective whitelists and sample-specific cell barcodes available at (https://github.com/morris-lab/BiddyetalWorkflow). As in the original publication, cells with >20 or <2 CellTags expressed were not considered for lineage assignment. Lineages were called on unfiltered data using a Jaccard similarity score of >0.7. Cell lineages were called on individual datasets for all analyses of a single time point. Data from Kimmerling et al.^21^ is GSE74923_L1210_CD8_processed_data.txt from GEO. Ground truth cell lineage information was extracted from the GSE74923_series_matrix.txt file. Data from Weinreb et al.^12^ was downloaded from (https://github.com/AllonKleinLab/paper-data/blob/master/Lineage_tracing_on_transcriptional_landscapes_links_state_to_fate_during_differentiation/README.md). Data from Jindal et al. was directly obtained from the authors in the form of Seurat objects including the unnormalized but filtered count matrix and a metadata file comprising the lineage information. All datasets were RPM normalized to 40,000 reads/cell. Data from Wehling et al.^23^ was directly obtained from the authors in the form of an unnormalized count matrix and the metadata file (available on GEO GSE167317 asGSE167317_CountMatrix_Seq5.csv, GSE167317_CountMatrix_Seq4.csv, GSE167317_Metadata_Seq5.csv, GSE167317_Metadata_Seq4.csv). Cells with under 100,000 reads were removed and data was normalized to 40,000 reads per cell. Lineage information was extracted from the metadata file. Data from Harmange et al. was downloaded from https://drive.google.com/drive/folders/1-C78090Z43w5kGb1ZW8pXgysjha35jlU?usp=sharing (accessed begin July 2022) in the form of 10×1_Filterd_BatchCor_unnorm_sctrans.rds from experiment one. Lineages were assigned using the corresponding script section in the file 10×1_r1_r2_Analysis_unorm_sctrans.Rmd. During filtering, cells with <3% of mitochondrial reads, and cells with <4000 reads were removed. Data was normalized to 40,000 reads/cell. Data from Simeonov et al.^42^ was obtained in the form of CellRanger output files for the lung metastasis scRNA-seq data of mouse 1 and 2 from GEO (GSE173958; GSM5283486 and GSM5283491). scRNA-seq data was normalized to 40,000 reads/cell. Lineage annotation was obtained from Mendeley data (DOI: 10.17632/t98pjcd7t6.1) in the form of “Barcodes-of-barcodes” files for all clones. This metadata was then filtered for the annotations of clones present in the scRNA-seq datasets of the lung metastases of mouse 1 and 2. Data from Bues et al.,^43^ was directly obtained from the authors in the form of a Seurat Object including a normalized count matrix with crypt and organoid annotation. Briefly, organoids were generated by sorting single Lgr5^+^ intestinal stem cells from dissociated organoids into a Matrigel matrix. After culture for 3, 4, 5, or 6 days, single organoids were hand-picked and dissociated individually before loading for scRNA-seq. Organoids are derived from 3 batches. Lgr5^+^ intestinal stem cells seeded can be derived from the same organoid. Crypts were collected over 5 batches of 3 pooled mice from 10 mm sections of the ileum. Crypts within the same batch can be derived from the same mouse but were several mm apart in the ileum. Data and scripts from Miller et al.^27^ were downloaded from https://github.com/vangalenlab/MAESTER-2021 and https://vangalenlab.bwh.harvard.edu/resources/maester-2021/ as TenX_CellLineMix_cells.rds, TenX_CellLineMix_All_mr3_maegatk.rds, TenX_CellLineMix_Seurat_Keep.rds (for the K562 dataset) and BPDCN712_Maegatk_Final.rds, BPDCN712_Seurat_Final.rds, 4.2_vois.txt (for the primary human bone marrow cells). Lineage assignment was performed as in the scripts 3.4_TenX_K562_clones.R and 4.3_variants_Of_Interest.R for K562 and bone marrow cells respectively. “GroupIDs” were then unlisted to extract the cell-barcode lineage correspondence. The count-matrix for the K562 dataset was extracted from the TenX_CellLine_Mix Seurat object by filtering for K562 cells. For the primary bone marrow cells, the BPDCN712 Seurat object was split out by replicate to generate the GEMLI input count matrices. For both cell types, lineages were filtered to be defined by a single informative mitochondrial variant. Data from Janesick et al.^44^ was downloaded from https://www.10xgenomics.com/products/xenium-in-situ/preview-dataset-human-breast. The scRNA-seq dataset was filtered to a > 1000 reads/cell and a mitochondrial fraction of 1-50%. Cell types were annotated through supervised machine learning. In brief, a UMAP was built upon a linear discriminant analysis (LDA; R package ‘MASS’ (v. 7.3-58.3) performed on log molecule numbers) which in turn was based on provided cluster annotations for Xenium data in situ sequencing data. Single-cell RNA-seq Chromium data was then projected onto this LDA-UMAP to assign cell types. Assignments were specific and unique. Finally, each cluster was assigned its cell type annotation through the assessment of marker genes provided in Janesick et al.^44^.

### ScRNA-seq data representation

ScRNA-seq data is represented as tSNE, UMAP, or SPRING graph. tSNE embeddings were generated using a custom function based on the tSNE R package (v. 0.1-3.1) on exonic, or both intronic and exonic reads. PCA was calculated on the 3,000 top variable expressed genes. Subsequently, a tSNE was computed on the PCAs top 10 components. SPRING graphs were generated from precalculated values in public datasets (HSPC data was represented as SPRING graph)). UMAPS (mESC, crypts, and organoid datasets) were generated in a standard Seurat analysis. Briefly, variable features were called using the FindVariableFeatures function (parameters selection.method =”vst”, and number of features = 2000), data was scaled using the ScaleData function on all genes, and a PCA was run using the RunPCA function on variable features. Subsequently, a UMAP was built using the RunUMAP function on the top 14 components.

### Assessment of gene expression similarity

Gene expression correlation within cell lineages or random samples for the whole transcriptome was calculated as Pearson correlation. Correlation distance is calculated as 1- the correlation value. To assess the similarity of related cells with respect to cell cycle, genes were classified as cell cycle-dependent or independent using a cell cycle assignment by the cyclone function within scran (v. 1.18.7). Dependence of gene expression on the cyclone assigned cell cycle phases (numerized) was tested using Hoeffding’s D statistics. Gene expression correlation was then calculated using only genes classified as cell-cycle dependent. To assess the similarity in the velocity of related cells, velocity analysis was performed using the velocyto package (v. 0.6). Velocity momenti were extracted for each cell, and their Pearson correlation was calculated. Complexity was defined as the number of genes expressed in a cell and the complexity range within cell lineages was calculated for analysis. For all metrics (transcriptomic similarity in all genes, in cell-cycle dependent genes, similarity in velocity momenti, and in complexity) values between related cells (all lineage sizes) and repeated (100x) random samples of cells of the same size were compared. For the scoring of transcriptomic similarity in symmetric and asymmetric lineages, symmetric and asymmetric lineages were defined based on entire lineages.

### Estimation of variation mediated by cell lineages

Assuming that the variation (here CV^2^) within each lineage is independent of cross-lineage effects, we built a linear model using the *stats* package (v. 3.6.2) to capture the relationship of the variation across lineages (variation of means of each lineage) and total variation (variation across all cells). We then generated mock lineages through the permutation of ground truth lineages and used our model to estimate the total variation in these control sets. Lineages of sizes 3–5 for mESCs and WM989 cells, of size 4 for CD8 and L1210, and of size 2 for all other cell types were considered. This ensured to inclusion of around 90% of the cells in each dataset.

### Memory-gene identification and categorization

We defined memory genes as genes with high variability (coefficient of variability squared; CV^2^) in mean gene expression in different cell lineages. We compared the lineage value to the distribution of values from repeated (20x) random size-matched samples of cells to calculate a p-value. Memory genes were defined as genes with a significantly (p < =0.05) higher variability between lineages as compared to random samples. To categorize memory genes into quantitative and qualitative memory genes, we used the *skewness* function of the R package *moments* (v. 0.14.1). We defined a quantitative memory gene as a gene with a skewness in expression level of under 3, and a qualitative memory gene, as a gene with a skewness in expression level equal or above 3. Memory genes were called on cell lineages of 3–5 (mESCs), 2 members (invasive tumor), or 2–5 members (all other cell types). All predicted cell lineages were used when calling memory genes on these. Other criteria to call memory genes resulted in highly overlapping gene sets (Fig. S6a, b). These additional memory gene selection criteria included a high correlation in gene expression within cell lineages and a low variability (CV^2^) in gene expression between cells of the same cell lineage (intra-lineage CV^2^; intraCV^2^). Furthermore, we analyzed marker genes of cell lineages using the Seurat FindMarker function with a range of *test.use* parameters (*bimod, roc, t* (T-test)*, negbinom, poisson, LR, MAST*). We then considered as memory genes the markers with a high sharing across cell lineages, using thresholds defined independently for each *FindMarker* run to optimize the Pearson correlation in gene expression within cell lineages. Finally, to identify memory genes using machine learning, we used common dimension reduction methods, mutual information maximizer (MIM), and ANOVA F-test feature selection (ANOVA). Other feature selection techniques frequently used in the literature were also investigated, but MIM and ANOVA outperformed all the other methods. For both, each gene was attributed a memory score either by computing the mutual information or ANOVA *F*-test of the gene expression across cells. To determine the ideal number of genes N to include in the final gene set, we predicted cell lineages using the top N genes with the highest memory score. Each of the resulting clusters was evaluated and the number of genes N with the best precision was chosen as the optimal number of memory genes. For the comparison of memory genes and variable genes in the Janesick et al.^44^ dataset, memory genes were called on lineages predicted at confidence level 50 with a lineage size parameter of 2–20 in each cell type independently. Genes were considered to be variable when having a residual to a least total square fit on mean expression and CV² above zero.

### GO-term enrichment analysis

GO-term enrichment analysis was performed on memory genes called using the lineage ground truth (derived from cellular barcoding, microfluidics, or sister cell picking), and on memory genes called using predicted cell lineages. The *topGO* R package (v. 2.42.0) was used with the GO category “biological process”. Memory genes and background (all genes detected) were considered to be binary. The top 10, 20, 100, or 500 enriched GOs from each set were visualized as indicated. Spearman rank correlation between GO-term enrichment values was calculated for memory genes called on-ground truth and predicted cell lineages. For GO-term enrichment of memory genes called based on predicted lineages in the Janesick data, GO-terms were further compared to expression-matched random gene sets. Enriched terms were significant (Fisher’s exact test, *p* < 0.001) and enriched over 100 expression-matched random gene sets (estimated *p* < 0.05). A selection of terms is shown in Fig. S22a and the full list can be found in Supplementary Data file 5.

### Gene selection for GEMLI predictions

Genes for cell lineage predictions were selected to enrich memory genes based on the quantiles of the mean and variability of gene expression. The variability of gene expression was defined as mean-corrected CV^2^ calculated in the form of the residual of the CV^2^ to a linear fit of CV^2^ and mean expression. The genes selected for lineage predictions are the 2% highest expressed genes, the 60% most variable among the 10% highest expressed genes, and the 10% most variable among the 40% highest expressed genes: (mean quantile >= 98) or (mean quantile >= 90 and variability quantile >= 40) or (mean quantile >= 60 & and variability quantile >= 90)). The selected genes are taken as input for the repetitive iterative clustering algorithm. The selected genes do only partly overlap with highly variable genes and cell type markers called during a standard Seurat-based scRNA-seq analysis (Figs. S4f and  S7f). For this comparison, highly variable genes were called as top 2000 variable genes with standard Seurat parameters. Cell type markers were called using Seurat’s FindMarkers function for each cell type present with at least 5 cells. Also using machine learning to select genes based on variability and mean gene expression did not further enrich memory genes (Fig. S7c). Briefly, different models were trained including logistic regression with L2 regularization (LR), support-vector machine (SVM) with the radial basis function (RBF) kernel, and a neural network. The mESC and Biddy et al. MEF datasets of 48 h and 72 h culture time (*n* = 1 and *n* = 6, respectively) were used for model training, the datasets of CD8, L1210 (*n* = 1 each), and HSPC (48 h; *n* = 22) were used for model validation. The mean expression outliers in each dataset were removed using the inter-quartile range (IQR) method. All the outliers were considered to be memory genes and were added in the final gene selection. Then, the data was normalized to a range between 0 and 1. Each dataset was preprocessed individually. The LR and SVM models were trained using their sklearn (v. 1.1.1) implementation. The neural network was optimized using the Pytorch framework (v. 1.11.0). To optimize the model, a balanced version of the binary cross entropy loss and accuracy was used to account for the fact that the proportion of memory genes is low in all datasets while most machine learning models assume that the data has approximately the same number of samples of both classes. To take this into account, a bigger weight was given to a misclassification of the minority class, the memory genes. To find the optimal hyperparameters for the LR and SVM a grid search and cross-validation were performed. Based on this, the L2 regularization was adjusted to 10^3^ and 10^5^ for LR and SVM respectively. For the neural network, cross-validation was deemed too computationally expensive, and the Optuna Python package (v. 2.10.0) was used to perform a random search for the optimal hyperparameters on training and validation set (Supplementary Data file 6). The best performing model was the neural network and its results are show in the Supplementary Figs.

### GEMLI cell lineage prediction algorithm

The GEMLI algorithm is a repetitive iterative clustering approach to predict cell lineages (Scheme in Fig. 2a, see Table S1 for a comparison to other clustering algorithms), taking a gene set as input. For de novo cell lineage predictions on scRNA-seq data without lineage annotation the gene set is selected based on mean gene expression and variability (see previous section). Also other gene sets can be used as input, notably memory genes when a ground truth lineage annotation is available (see below). During each iterative clustering, the input gene set is randomly subsampled to 75% (sampling value parameter; see section on GEMLI parameters below). This gene set subsample is then used to cluster the cells of the dataset iteratively as follows. During the first iteration, two clusters (cluster cut parameter; see below) are generated using *cutree* function from the dendextend (v.1.16.0) R package and the *hclust* hierarchical clustering function of the R package stats (v.4.0.2) on the gene expression correlation distance using the agglomeration method ward.D2. In every subsequent iteration round, the cells of each previously defined cluster are clustered again into two clusters using the same procedure. The iteration is ended for clusters meeting a predetermined size of 2–3 cells (lineage size parameter, see below). This results in a cell-by-cell matrix indicating for each cell-pair if it did cluster together in the final cluster of 2–3 cells. This iterative hierarchical clustering represents one-cell lineage prediction. It is repeated 100 times (repetition number parameter, see below) to generate a confidence level for the lineage predictions. More in detail, the cell-by-cell matrices generated in the 100 repetitions of the iterative hierarchical clustering are summed into a confidence level cell-by-cell matrix. Two cells ending up in the same cluster in 30 of the 100 predictions will here for example receive the value of 30. Setting a threshold on the confidence level can then be used for lineage assignment. Cell lineages are assigned as cell groups of which members are clustering together above a confidence level threshold. Lineage assignments are scored against the ground truth lineages (barcode, microfluidic, or sister-cell picking) on precision (true positives (TP)/(TP+ false positives (FP))), sensitivity (TP/(TP+ false negatives (FN))) and false positive rate (FPR; FP/(FP+ true negatives (TN))). All are calculated for each confidence level X by comparing the predictions with a confidence level >=X to the ground truth cell lineage relationships. Thereby precision, sensitivity and FPR are calculated on cell pairs. For the majority of datasets, also AUC values were calculated using the R package Metrics (v. 0.1.4) by the confidence level of predictions as a probability value.

### Comparison of GEMLI to other lineage assignment approaches

To test the performance of GEMLI predictions, characteristics-based gene selection was compared to other starting gene sets, as all genes and memory genes called using the lineage ground truth (from cellular barcoding, microfluidics, or sister cell picking). Further, different memory gene definitions (see above) and Seurat’s highly variable genes (see above) were tested as input gene sets. Finally, memory gene enriched gene sets selected based on mean expression and variability using a neural network (see above) were tested in predictions. GEMLI predictions had a slightly lower precision and sensitivity than predictions using memory genes, but performed better than all genes (Fig. S8). FPR was comparable between all three genesets (GEMLI, memory genes, all genes) and consistently very low (<1%) at confidence levels >=50. Other memory gene definitions did not improve prediction performance (Fig. S6c–h). Also, gene selection based on a neural network did not improve lineage predictions as compared to GEMLI (Fig. S9d). Seurat’s highly variable genes likewise performed worse than the GEMLI gene selection (Fig. S9f). Furthermore, the use of a memory gene set called using the ground truth lineage information in one dataset, did also not allow for improved predictions in other datasets of the same or related/other starting cell type and culture condition (Fig. S9e). Finally, GEMLI performance was also compared to other lineage assignment approaches, namely random cell pair assignment and kNN-clustering-based lineage assignments. GEMLI predictions on randomly lineage-assigned cells had as expected a very low precision and sensitivity and a high FPR (Fig. S9a). Furthermore, cell pairs generated using kNN-clustering with different inputs (Seurat top 2000 variable genes, top 14 components of a PCA on these genes, UMAP on this PCA) resulted in a very low precision, comparable sensitivity, and higher FPR than GEMLI predictions (Fig. S9b, c).

### GEMLI predictions for symmetric and asymmetric lineages and different lineage sizes

Scoring predictions for symmetric (one cell type) and asymmetric (several cell types) lineages were performed in three ways. First, only two-cell lineages were scored, which can be unequivocally defined as symmetric (both members of one cell type) and asymmetric (each member of another cell type). Second, lineages of all sizes were scored for which symmetry was defined considering all cell members. Lineages with all members in one cell type were here considered symmetric. Lineages in which any members were part of two different cell types were considered to be asymmetric. Third, cell pairs were scored on the entire prediction matrix by taking pairs where both cells are of the same cell type as symmetric lineage, and pairs where both cells are of a different cell type as asymmetric lineage, respectively. For lineage size analyses the prediction matrix was stratified to only score pairs in which one or both cells are members of a lineage of the indicated ground truth lineage size.

### KNN-clustering-based lineage assignments for comparison to GEMLI predictions

For kNN analysis a standard Seurat pipeline was used. Data was normalized and the 2000 most variable features were extracted using default parameters. The data was then scaled, a PCA was performed on previously defined variable genes and a UMAP was conducted on the first 14 principal components. The kNN analysis was then performed using the dbscan package (v. 1.1-11) with *k* = 1 or 2 as indicated, on either the scaled data for variable features, the 14 first principal components or the UMAP embedding. Pairs of nearest neighbors were used as the lineage for comparison with GEMLI predictions.

### GEMLI prediction algorithm parameters

Several parameters can be set during GEMLI predictions: (1) the sampling value (which fraction of genes is selected for each repetition of the iterative hierarchical clustering), (2) the number of repetitions of the iterative clustering (based on which the confidence level is calculated), (3) the number of clusters in which clusters are split during each iteration (“cluster cut”) and (4) the size of clusters at which clustering iterations are stopped (“lineage size parameter”). The default values used throughout the main figures are a sampling value of 75%, 100 repetitions, splitting clusters into 2 during each iteration, and lineage size parameter of 2–3 cell members unless otherwise indicated. The influence of changes in single parameters on predictions was tested on the unique mESC, CD8, L1210, and one HSPC, WM989, and HSC dataset (Figs. S10 and  S12). A lower sampling value increased precision while decreasing sensitivity (Fig. S10a, b). When the number of clusters into which each cluster is split is higher than the maximal lineage size to be predicted, precision values increase and sensitivity decreases (Fig. S10c, d). The number of repetitions did only had a small effect on precision and sensitivity values, with higher repetition numbers increasing precision and decreasing sensitivity of the predictions (Fig. S12e, f). Also, the lineage size parameter, meaning the size of clusters at which clustering iterations are stopped influences both precision and sensitivity. Precision will be high for predictions with a lineage size parameter of 2–3 until the average size of ground truth lineages is present (Fig. S12). FPR stayed low throughout. Only a size parameter greatly exceeding the average ground truth lineage size (Fig. S12c–e) can increase FPR to values > 1%, but still <5%. For predictions of multicellular structures (intestinal crypts and organoids) for which related cells are in spatial proximity to each other, the cluster stability of lineages predicted at increasing lineage size parameters, (cluster stability calculated using the sc3 package within the R package clustree (v.0.5.0)), reflected well the ground truth lineage size and plateaued when reaching the maximal ground truth lineage size present. In contrast, for predictions in datasets with primarily small lineages without a spatial proximity or confinement, sc3 cluster stability of predictions increased gradually with increasing lineage size parameter and did not plateau. This allowed for a de novo estimation of the lineage size until which prediction could be performed with high precision in datasets with multicellular structures (fig. S19). Depending on the aim of cell lineage predictions and the dataset characteristics (see next section), parameters and confidence level threshold can thereby be adjusted to increase precision or sensitivity respectively.

### Influence of sequencing depth and cell number on GEMLI predictions

To estimate the influence of sequencing depth and dataset size on lineage predictions, GEMLI predictions were performed after downsampling the number of reads or cells in the mESC dataset, and one MEF and HSPC dataset (Fig. S15 and Supplementary Data file 1). For read downsampling, all reads of a given cell were vectorized, and subsequently, a fraction (66%, 50%, 33%, 10%) of these reads were sampled. Cells were subsampled separately to 66%, 50%, 33%, and 10% of the initial cell numbers. Cells without lineage assignment or being the only member of a cell lineage were subsampled directly to the desired fraction. Cells that were members of cell lineages with several members were subsampled together with their respective cell lineage members. Sensitivity of lineage predictions decreased with decreasing sequencing depth but the precision of lineage predictions stayed high above values of 5000–8000 reads/cell as commonly recommended for available scRNA-seq technologies^37–39^ (Fig. S15a, b). Subsamples with fewer cells generally had a slightly better precision and sensitivity, especially for very low read counts (Fig. S15c, d). FPR stayed <1% for all conditions.

### Cell fate decision analysis

To assess how well GEMLI can recapitulate the prevalence of different types of cell fate decisions, the HSPC (N = 44) and WM989 (N = 8) datasets were analyzed. HSPCs datasets are derived from cells cultured in myeloid differentiation conditions and are annotated according to their cell type as basophil (Baso), eosinophil (Eosino), erythroid, lymphoid (Lymph), megakaryocyte (Meg), monocyte (Mono), neutrophil (Neutro), mast cell, plasmacytoid dendritic cell (pDC (1/2)), migratory dendritic cell (Ccr7^+^), classic dendritic cell (cDC) or undifferentiated (Undiff) cells (published metadata^12^). Only certain cell type combinations of related cells exist and all have a different prevalence, meaning that HSPCs are restricted in their fate decisions. For each possible cell type combination (for example, erythroid-eosinophil or undifferentiated-mast cell) the number of cell pairs in barcode and GEMLI lineages was determined and summed for all datasets. WM989 cells are annotated as drug-susceptible or drug-resistant (published metadata^4^). Members of each cell lineage are either entirely in one of these two states (symmetric cell pairs) or distributed between these two states (asymmetric cell pairs), with the latter implying that cells recently switched fate. We compared the number of barcodes- and GEMLI-predicted related cell pairs being entirely drug-susceptible, primed, or distributed between the two states. Both for HSPC and WM989 datasets, also DEGs called between different related asymmetric and symmetric cell pairs were compared in barcode and GEMLI lineages. DEG was called using the *FindMarkers* function of Seurat with parameters min.pct=0.05 (HSPC) or 0.25 (WM989) and logfc.threshold=0.1. For HSPCs, not all comparisons of cell type pairs allowed DEG identification. To compare GEMLI and barcode lineages, the Spearman rank correlation between enrichment scores was calculated. The performance of GEMLI in recapitulating the prevalence of lineages and encompassing different cell types (HSPC) of states (WM989) was also compared to random cell lineage assignments for HSPC and WM989 datasets. For the HSPC datasets, it was further compared to a kNN-clustering based lineage assignment. Likewise, the performance of GEMLI in recovering DEG specific to cells in lineages composed of specific cell types or states was also compared to random cell type and kNN-based lineage assignments as follows. For the DEG analysis in Fig. 3e–d the correlation between the enrichment of DEG in ground truth lineages and predicted lineages for which members were exchanged against random cells of the same cell type was calculated (Fig. S17e, f). For the HSPC datasets, DEG analysis was also performed for random cell samples and kNN-based lineage assignments in all datasets (Fig. S17a, d). Cell-type combinations predicted by GEMLI were scored and compared. Trajectory analyses were performed using the *scran* (v. 1.28.2) and *slingshot* (2.8.0) packages. Analyses were performed on the 200 (HSPC) or 2000 (WM989) most variable genes on a UMAP with cluster annotation based on a nearest-neighbor graph. Cells in transition was identified based on pseudo-time as those cells of each type that are located close to the transition point between undifferentiated cells and neutrophil cells (HSPC) or drug-susceptible and primed cells (drug-resistant; WM989) respectively. To analyze cell fate decisions in the human breast cancer cell subsets of the Janesick et al.^44^ dataset, DEG were called as described above for all cell pairs being members of lineages predicted with lineage size parameter 2–20 cells at confidence level 50 annotated as encompassing only DCIS or invasive tumor cells (symmetric DCIS and symmetric invasive tumor lineages respectively) or being members of lineages encompassing both cell types (asymmetric lineages). For the latter category, only DCIS or invasive tumor cells were considered when indicated.

### Datasets considered for analysis

For datasets considered in each figure and analysis see Supplementary Data file 1. For all figures in which one dataset of seven cell types is represented the unique mESC, CD8, L1210, as well as the MEF dataset BIDDY_D0_2, the WM989 dataset WM989_well1, the HSC dataset HSC_seq2, and the HSPC dataset LK_D2_exp1_library_d2_2 are represented. To show a dataset encompassing a large number of asymmetric lineages, the dataset LK_D4_well1_exp1_library_d4_1_2 is used in Fig. 3e. For other panels see Supplementary Data file 1.

### Statistics and reproducibility

A two-sided Mann–Whitney *U*-test, which does not require normally distributed data, was used to test statistical significance when indicated. No statistical method was used to predetermine sample sizes. No data were excluded from the analyses. The experiments were not randomized. The investigators were not blinded to allocation during experiments and outcome assessment.

### Reporting summary

Further information on research design is available in the Nature Portfolio Reporting Summary linked to this article.

### Supplementary information

Supplementary Material

Peer Review File

Description of Additional Supplementary Files

Supplementary Data file 1

Supplementary Data file 2

Supplementary Data file 3

Supplementary Data file 4

Supplementary Data file 5

Supplementary Data file 6

Reporting Summary

### Source data

Source Data

## Data Availability

All relevant data supporting the key findings of this study are available within the article and its Supplementary Information files. CGR8 mESC scRNA-seq data generated in this study has been deposited on GEO under accession code GSE226169. The MEF datasets used in this paper are available at GEO under accession codes GSE99915, The CD8 and L1210 cell datasets used in this paper are available at GEO under accession code GSE74923. The HSC datasets used in this paper are available at GEO under accession code GSE167317. The WM989 cell datasets used in this paper are available at GEO under accession code GSE237228. The pancreatic cancer datasets used in this paper are available at GEO under accession code GSE173958. The intestinal crypt and organoid datasets used in this paper are available at GEO under accession code GSE148093. The HSPC datasets used in this paper are available at GEO under accession code GSE140802. The human bone marrow and K562 cell datasets used in this paper are available at GEO under accession code GSE182685. The human breast cancer datasets used in this paper are available at GEO under accession code GSE243280. Source data are provided with this paper^50^.

## Code availability

The GEMLI R package and its documentation are available on GitHub at https://github.com/UPSUTER/GEMLI. A *Zenodo* repository has been created for the GitHub GEMLI package v1.0.0 under doi:10.5281/zenodo.10673334^51^. The code to produce all main figures of the paper is available on *Zenodo* doi: 10.5281/zenodo.10581737^52^.
