# Author Correction: Gene-expression memory-based prediction of cell lineages from scRNA-seq datasets

**DOI:** 10.1038/s41467-024-48978-8

**Published:** 2024-06-04

**Authors:** A. S. Eisele, M. Tarbier, A. A. Dormann, V. Pelechano, D. M. Suter

**Affiliations:** 1 Ecole Polytechnique Fédérale de Lausanne, School of Life Sciences, Institute of Bioengineering, Lausanne, Switzerland; 2 Science for Life Laboratory, Department of Microbiology, Tumor and Cell Biology, Karolinska Institute, Solna, Sweden

**Keywords:** Computational biology and bioinformatics, Epigenetic memory, Gene expression profiling, Computational models, Gene ontology

Correction to: *Nature Communications* 10.1038/s41467-024-47158-y, published online 29 March 2024

In the Acknowledgements section of this article the grant number relating to Swiss National Science Foundation given for D.M.S. was incorrectly given as grant#CRSK-3_195097 and should have been grant#CRSII5_189910. The original article has been corrected*.*

