# Supplementary material for: Gene-expression memory-based prediction of cell lineages from scRNA-seq datasets
Source: Nat Commun. 2024 Mar 29;15:2744. doi: 10.1038/s41467-024-47158-y (PMC10980719; doi:10.1038/s41467-024-47158-y)
Supplement: Supplementary file 1 — Supplementary Material [file 41467_2024_47158_MOESM1_ESM.pdf]

Supplementary Materials for

Gene-expression memory-based prediction of cell lineages from scRNA-seq datasets

A.S. Eisele[1,*,†], M. Tarbier[2,†], A.A. Dormann[1], V. Pelechano[2], D.M. Suter[1,*]

†These authors contributed equally to this work.
*Corresponding authors: almut.eisele@epfl.ch and david.suter@epfl.ch.

This PDF file includes:

Figs. S1-S22
Table S1-2

Other Supplementary Material for this manuscript includes the following:

Supplementary Data files 1-6 (Excel files) and a Source Data file.

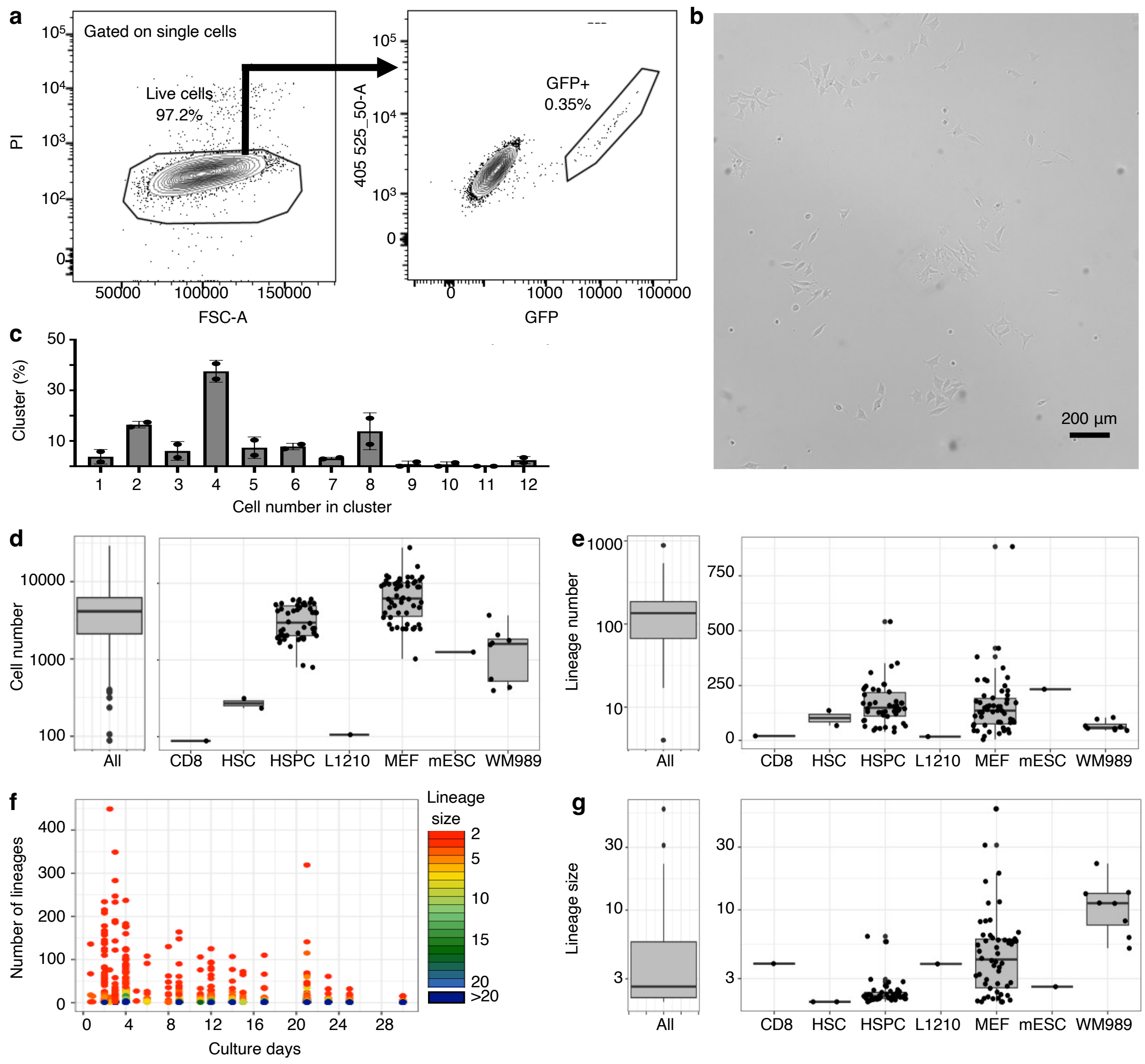

fig. S1: Overview of lineage-annotated scRNA-seq datasets. (**a**) Barcoded mESC cells were sorted as single, live GFP$^+$ cells into two wells of a 96-well plate. (**b**) Brightfield image of mESCs after 48h of culture before collection for scRNA-seq. (**c**) Distribution of cluster sizes of mESCs after 48h of culture (two wells) in a separate experiment counted manually in microscopy images. mESC were seeded at a low density to keep related cells clustered. Error bars: data range. (**d**) Number of cells across all (left) datasets and split by cell type (right; datasets of mESC, CD8, L1210 (n=1 for each), MEF (n=54), HSPC (n=45), HSC (n=2), WM989 (n=8)). (**e**) Number of lineages in all (left) datasets and split by cell type (right) as in (d). (**f**) Overview of the number of lineages of specific sizes (color) by culture time across all datasets as in (d). (**g**) Average lineage size for all (left) datasets and split by cell type (right) as in (d). For all boxplots: intervals between the 25th and 75th percentile and median (horizontal line). Error bars: 1.5-fold the interquartile range or the closest data point when no data point is outside this range. Source data are provided as a Source Data file.

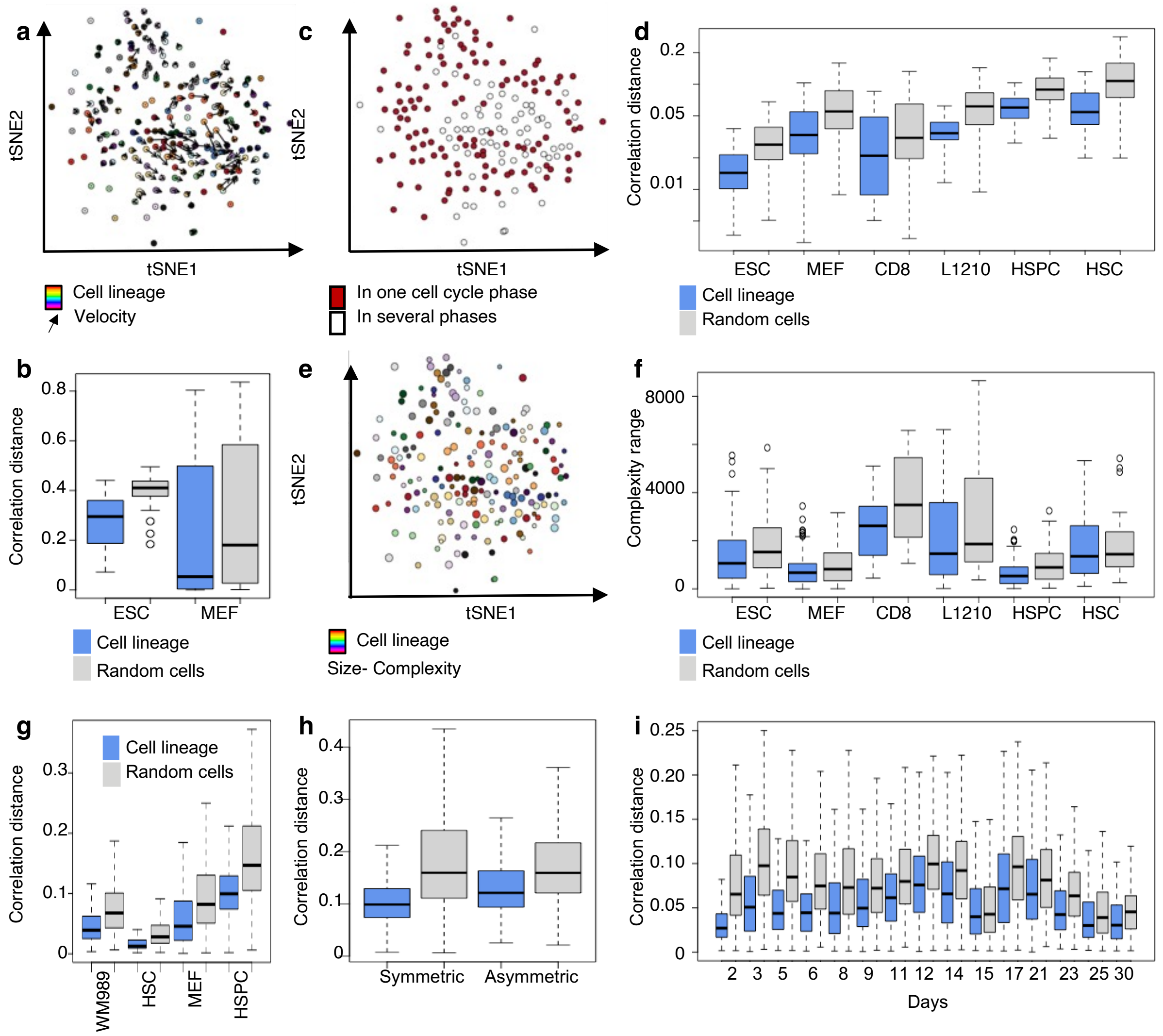

fig. S2: Similarity of cell lineages in scRNA-seq data within and across cell types. (**a**) tSNE embedding of a PCA on exonic and intronic data of the lineage-annotated (colors) mESC dataset (n=1) including velocity vectors (arrows). **(b)** Comparison of correlation distance in exonic and intronic gene expression for cells of the same cell lineage and randomly sampled cells in mESC and MEF datasets (n=1 for each). (**c**) tSNE embedding of the mESC dataset as in (a) coloring according to the completeness of each lineage in one cyclone-assigned cell-cycle phase. **(d)** Correlation distance in cell cycle-dependent gene expression in related and randomly sampled cells for the indicated cell types (n=1 dataset for each). (**e**) tSNE embedding of the mESC dataset as in (a) with size indicating the complexity (number of expressed genes) quantile of each cell. **(f)** Quantification of similarity in complexity range of related and randomly sampled cells for the indicated cell types (n=1 dataset for each). **(g)** Boxplot of the correlation distance in gene expression for related cells and randomly sampled cells (100 repetitions) in all datasets of the indicated cell types (n=datasets; n=8 for WM989, n=2 for HSC, n=14 for MEF, n=44 for HSPC). (**h**) Correlation distance as in (g) for lineages encompassing a single cell type (symmetric) or several cell types (asymmetric) in the HSPC datasets of day 4 (n=22 datasets). (**i**) Correlation distance as in (g) for different timepoints across a 30-day MEF reprogramming time course (n=4,10,2,2,4,4,2,6,2,2,2,8,2,2,2 datasets, respectively, for indicated days within the time course). For all boxplots: intervals between the 25th and 75th percentile and median (horizontal line). Error bars: 1.5-fold the interquartile range or the closest data point when no data point is outside this range. Source data are provided as a Source Data file.

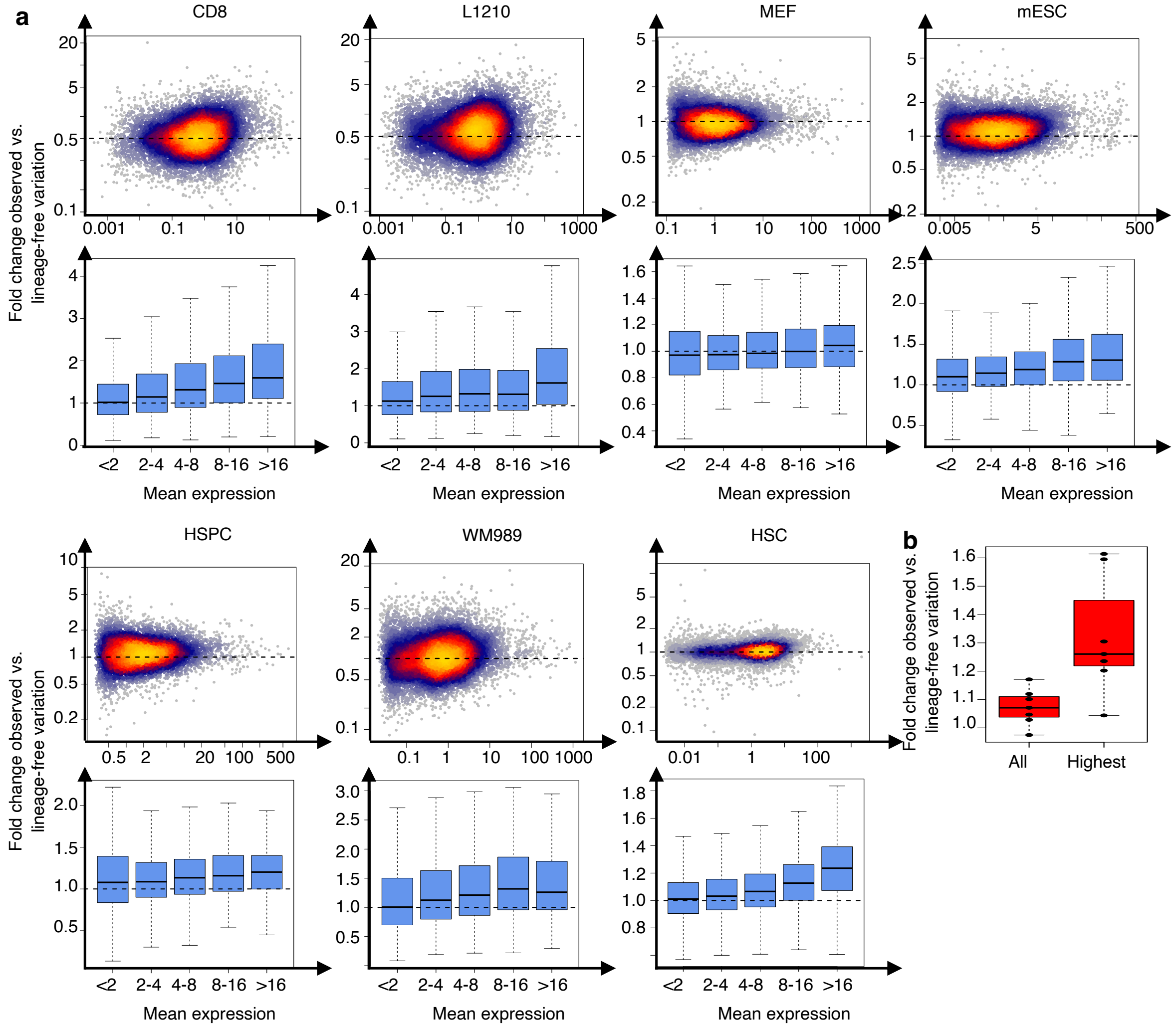

fig. S3: Influence of lineage-dependent gene expression on gene expression variability. (**a**) Scatterplots and boxplots of the fold change of observed vs. lineage-free variation as factor of mean gene expression in different datasets as indicated (n=1 dataset for each cell type). (**b**) Fold change of observed vs. lineage-free variation for all and the highest expressed transcripts for bins and datasets as in (a) (n=7). Boxes as in fig. S2. Source data are provided as a Source Data file.

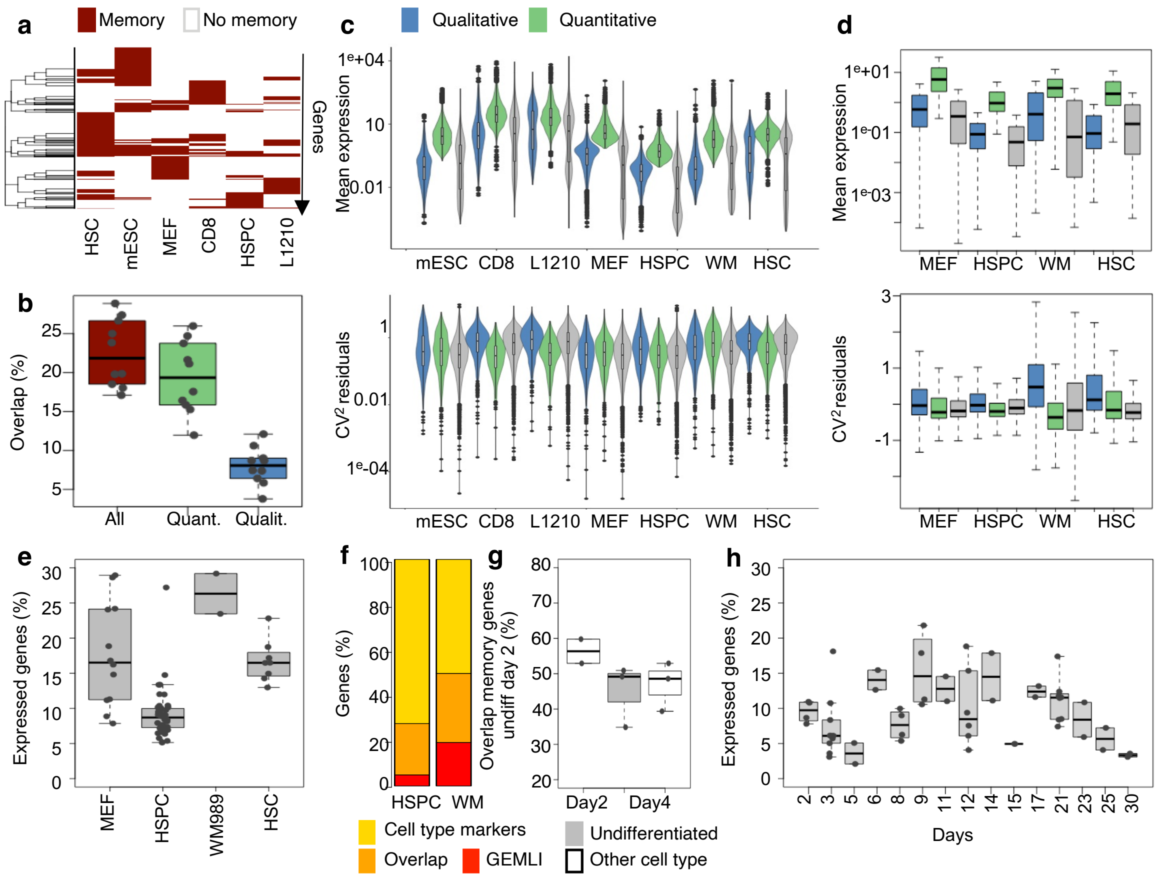

fig. S4: Characteristics and sharing of memory genes within and across cell types. (a) Memory genes in six different murine cell types as indicated (n=1 dataset in each cell type). (**b**) Pairwise overlap of memory genes between datasets as in (a). (**c**) Expression levels (top) and mean-corrected CV^2 (bottom) of the different gene categories across cell types for one dataset each. Boxes as in fig. S2 with an overlaid density plot. (**d**) Expression level (top) and mean-corrected CV^2 (bottom) of the different gene categories as in (c) for all datasets of the indicated cell types (n=datasets; n=8 for WM989, n=2 for HSC, n=44 for HSPC, n=14 for MEF). **(e)** Percentage of memory genes in expressed genes in the indicated datasets as in (d). (**f**) Mean percentage of genes in GEMLI gene selection and Seurat's cell type markers that are unique or overlapping in HSPC (n=44), and WM989 (n=8) datasets. (**g**) Overlap of memory genes of undifferentiated cell lineages (undiff) at day 2 of HSPC differentiation with memory genes of undifferentiated cell lineages, or lineages being composed of other cell types (other cell type) at day 2, and 4 (three time course experiments with n=4,3,6 and n=6,4,12 datasets at day 2 and 4, respectively). Memory genes of all datasets for a given experiment are considered. The overlap is given as the percentage of the later time point memory gene set being shared with memory genes of undifferentiated cell lineages of day 2. (**h**) Percentage of memory genes in expressed genes for different timepoints across a 30-day MEF reprogramming time course (n=4,10,2,2,4,4,2,6,2,2,2,8,2,2,2 datasets, respectively, for indicated days within the time course). Boxplots as in fig. S2. WM989 is abbreviated as WM in some panels. Source data are provided as a Source Data file.

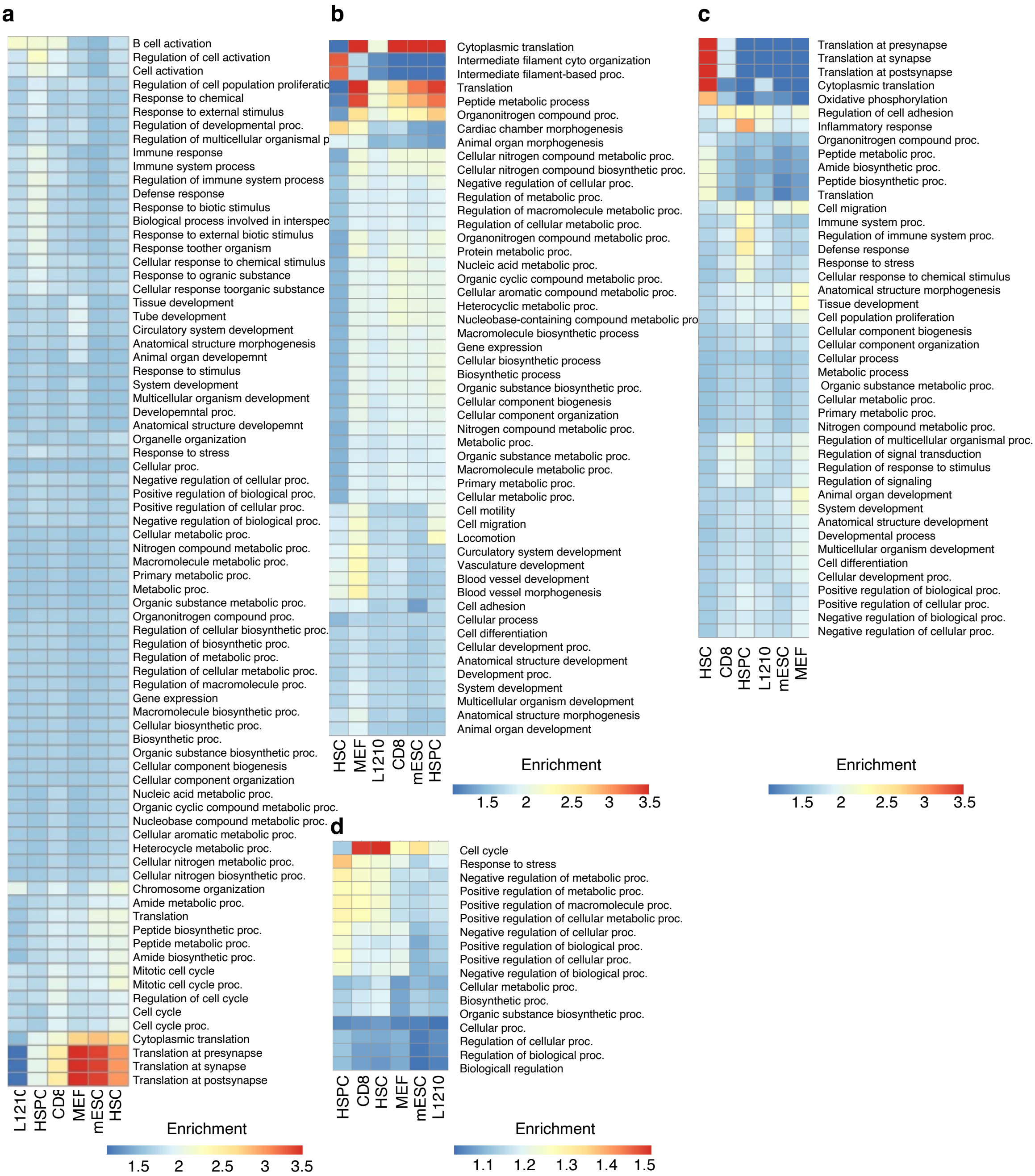

fig. S5: Memory gene GO-term enrichments. (**a-c**) Heatmaps of combined top 20 GO-term enrichment in (a) all memory genes, (b) quantitative and (c) and combined top 10 GO-term enrichment in qualitative memory genes of different murine cell types as indicated (n=1 dataset for each cell type). Note that a high overlap of top20 GO-terms in quantitative memory genes results in fewer represented GO terms. (**d**) Heatmap of the highest shared top 100 GO-terms of high memory genes in the datasets as in (a). Proc.: process. Source data are provided as a Source Data file.

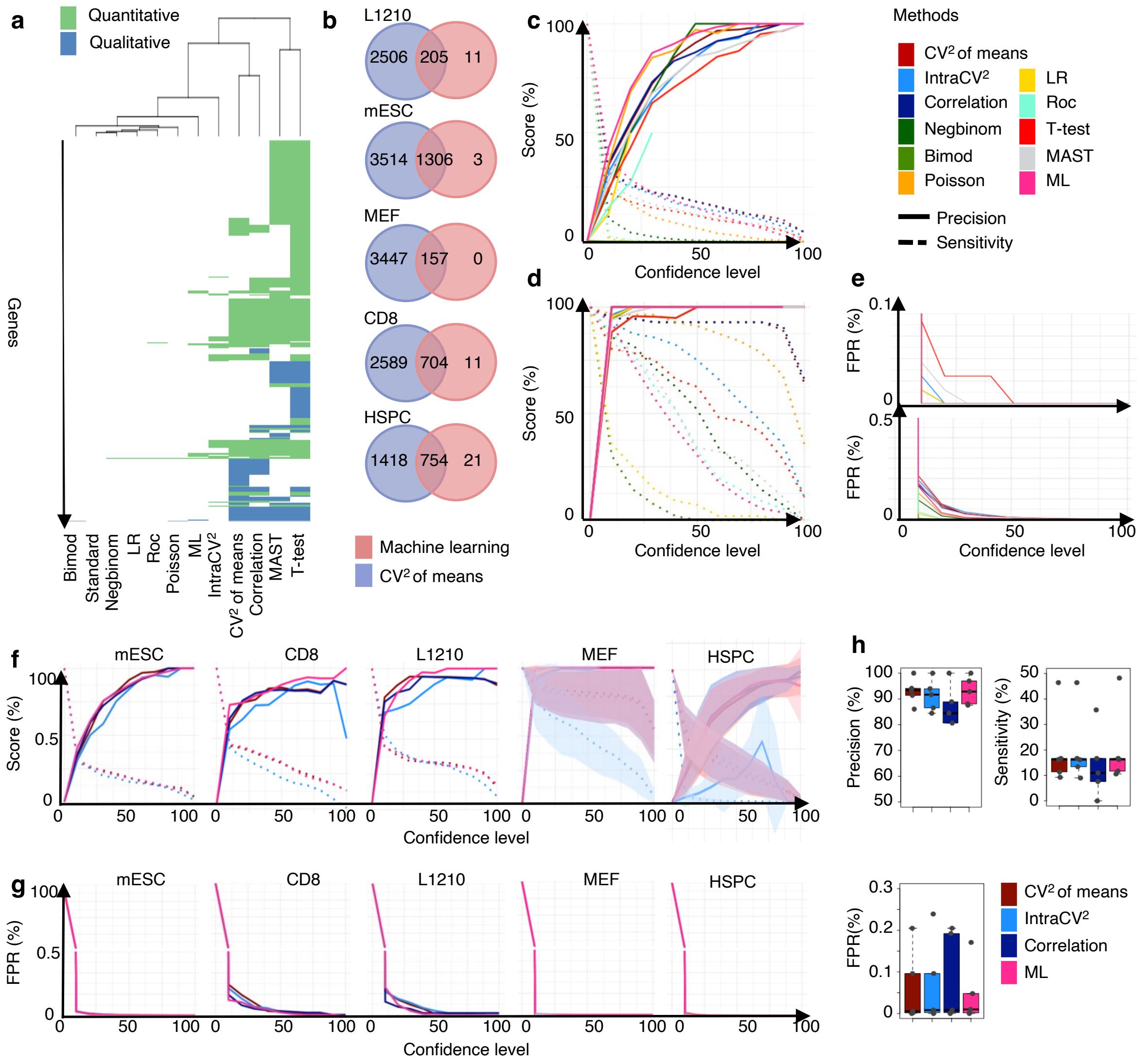

fig. S6: Comparison of different memory gene definitions for maximal predictive power. (**a**) Heatmap showing the sharing of memory genes called using different methods in the mESC dataset. Memory genes are selected based on a high correlation of gene expression within cell lineages (correlation), a small intra-cell lineage variability (intraCV^2), a large variability across means of cell lineages (CV^2 of means), are marker genes for cell lineages found using Seurat's FindMarker() function (Bimod, Standard, Negbinom, LR, Roc, Poisson, MAST, T-test), or have been selected using a machine learning (ML) approach. (**b**) Overlap in the Machine learning generated memory gene sets (red) and the CV^2 of means-based memory gene sets (blue) in the indicated cell types (n=1 dataset for each cell type). (**c**) Precision-sensitivity curves for predictions in the mESC dataset using memory genes defined using different methods (colors) as in (a) as input geneset (Precision=line; sensitivity=dotted line). (**d**) Precision-sensitivity curve for predictions on one MEF dataset as in (c). (**e**) FPR curve for predictions as in (c-d) for the mESC dataset (bottom) and MEF dataset (top). (**f**) Precision-sensitivity curves as in (e) for five cell types (n=datasets; n=1 for mESC, CD8, L1210, n=6 for MEF, n=20 for HSPC) for the four best performing methods to call memory genes (colors as in (c); line=mean precision, dotted line=mean sensitivity; shade= S.D.). (**g**) FPR curve for datasets and methods as in (f). (**h**) Boxplots of precision, sensitivity, and FPR for predictions as in (f) at confidence level 50 for the datasets as in (b; n=1 dataset for each cell type). Boxplot as in fig. S2. For abbreviations of memory gene definitions/selection methods see Methods. Source data are provided as a Source Data file.

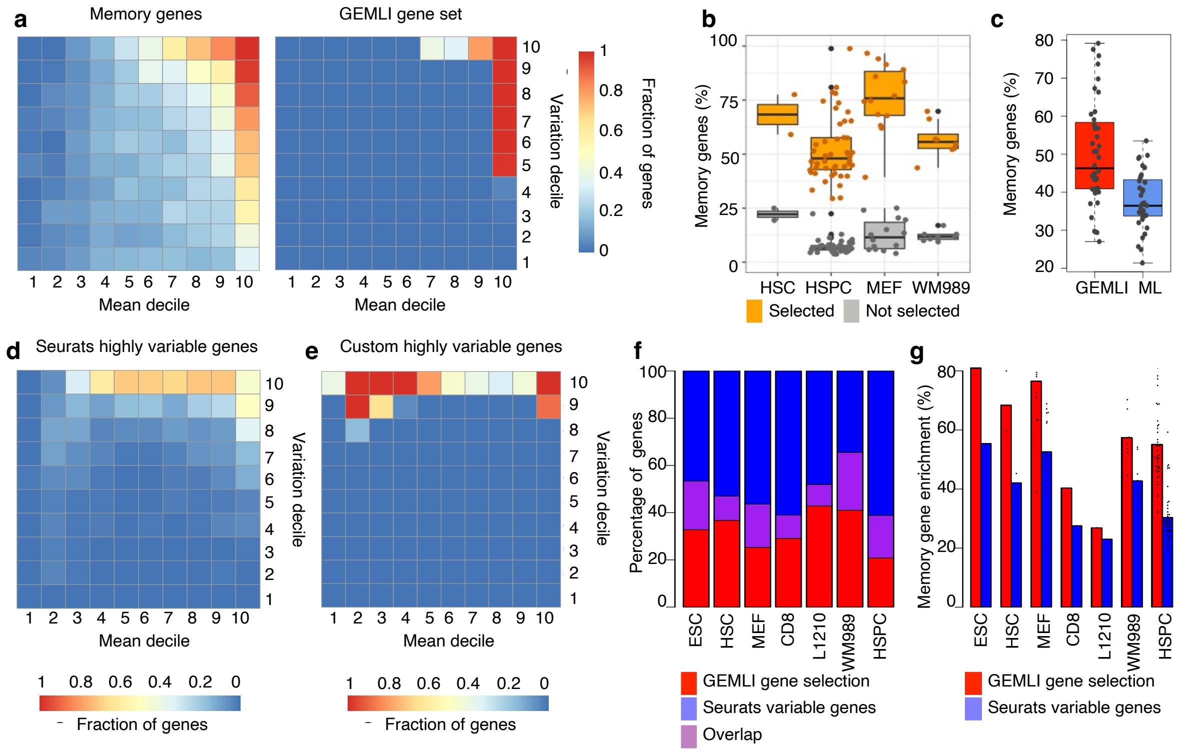

fig. S7: Enrichment of memory genes by GEMLI. (a) Abundance as a function of mean and variation deciles for memory genes (left) and the GEMLI gene set (right) on the mESC dataset. (**b**) Percentage of memory genes in the GEMLI gene set in the indicated cell types (n=datasets; n=2 for HSC, n=45 for HSPC, n=6 for MEF, n=8 for WM989). (**c**) Percentage of memory genes selected by GEMLI and a neural network (ML) based on variability and mean expression across 40 datasets of several cell types (n=1 for mESCs, CD8, L1210, n=2 for HSC, n=6 for MEF, n=8 for WM989, n=21 for HSPC). (**d**) Abundance as a function of mean and variation deciles as in (a) for Seurat's highly variable genes called on the mESC dataset. (**e**) Abundance as a function of mean and variation deciles as in (a) for a custom selection of highly variable genes called on the mESC dataset. (**f**) Percentage of genes in Seurat's highly variable genes and the GEMLI gene selection that is unique or overlapping. The mean over datasets is shown (n=1 for mESCs, CD8, L1210, n=6 for MEF, n=44 for HSPC, n=8 for WM989). (**g**) Fraction of memory genes recovered in Seurat's variable genes and by GEMLI. Datasets as in (f). Boxplots as in fig. S2. Source data are provided as a Source Data file.

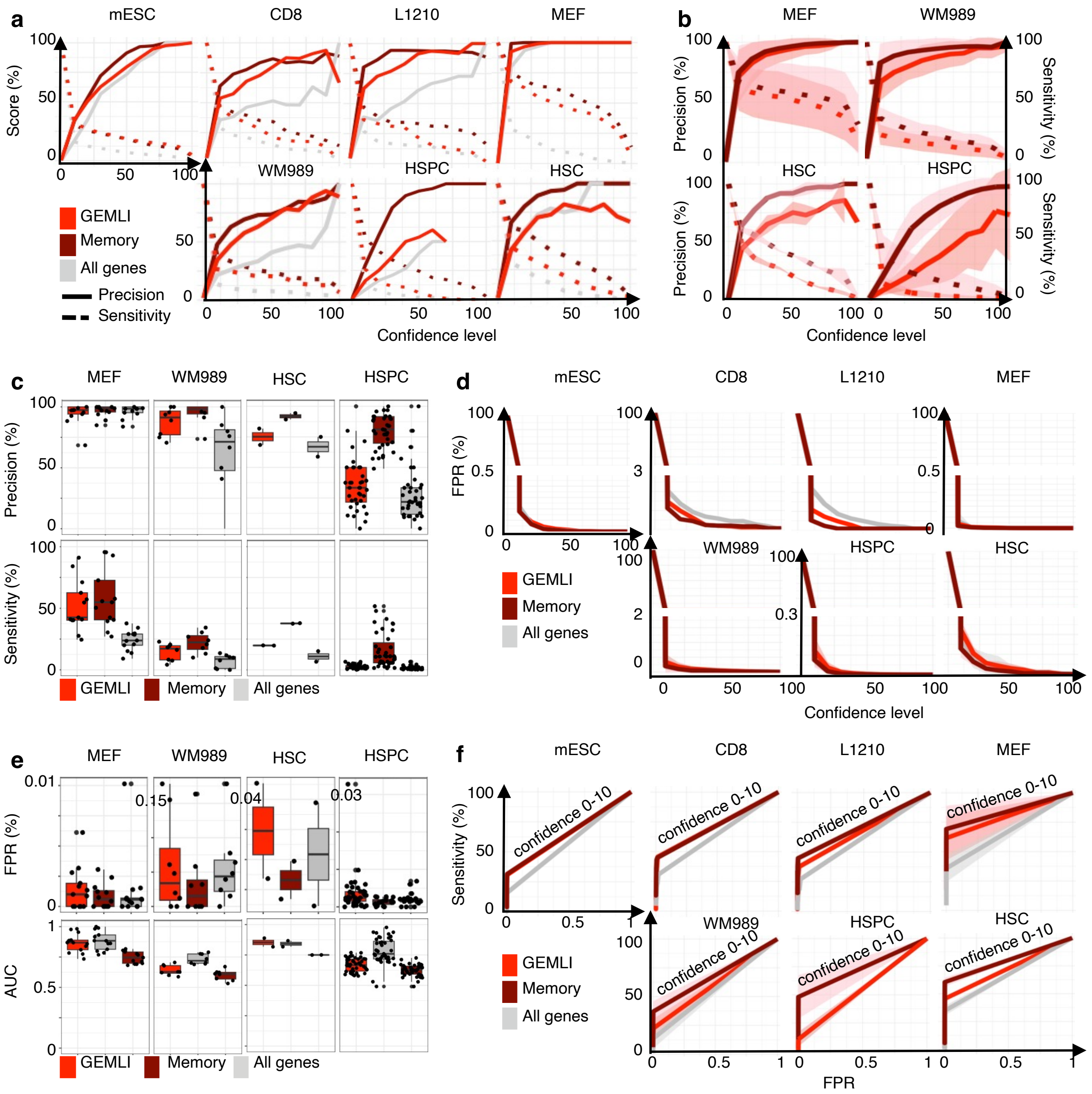

fig. S8: GEMLI lineage predictions performance in different cell types. (**a**) Precision-sensitivity curves of lineage predictions using all genes, memory genes called using the ground truth lineages, or genes selected by GEMLI in 7 cell types (n=1 dataset for each). (**b**) Precision-sensitivity curves of lineage predictions as in (a) in all datasets of the indicated cell types (n=14 for MEF, n=8 for WM989, n=2 for HSC, n=44 for HSPC). Line: mean; shades: S.D. (**c**) Boxplot of precision (top) and sensitivity (bottom) for lineage predictions across datasets as in (b) using all genes, memory genes, or GEMLIs gene selection as input at confidence level 50. (**d**) False-positive (FPR) curve of lineage predictions using all genes, memory genes called using the ground truth lineages, or genes selected by GEMLI in 7 cell types (n=datasets; n=1 for mESC, CD8, L1210, n=14 for MEF, n=8 for WM989, n=44 for HSPC, n=2 for HSC). Line: mean; shades: S.D. **(e)** Boxplots for datasets as in (c) of the FPR (top; confidence level 50) and AUC values (bottom) for lineage predictions using all genes, memory genes called using the ground truth lineages, or genes selected by GEMLI. **(f)** ROC curves for GEMLI predictions across cell types and datasets as in (d). The parts of the ROC curve corresponding to GEMLI predictions at confidence values of 0-10 are indicated. Boxplots as in fig. S2. Source data are provided as a Source Data file.

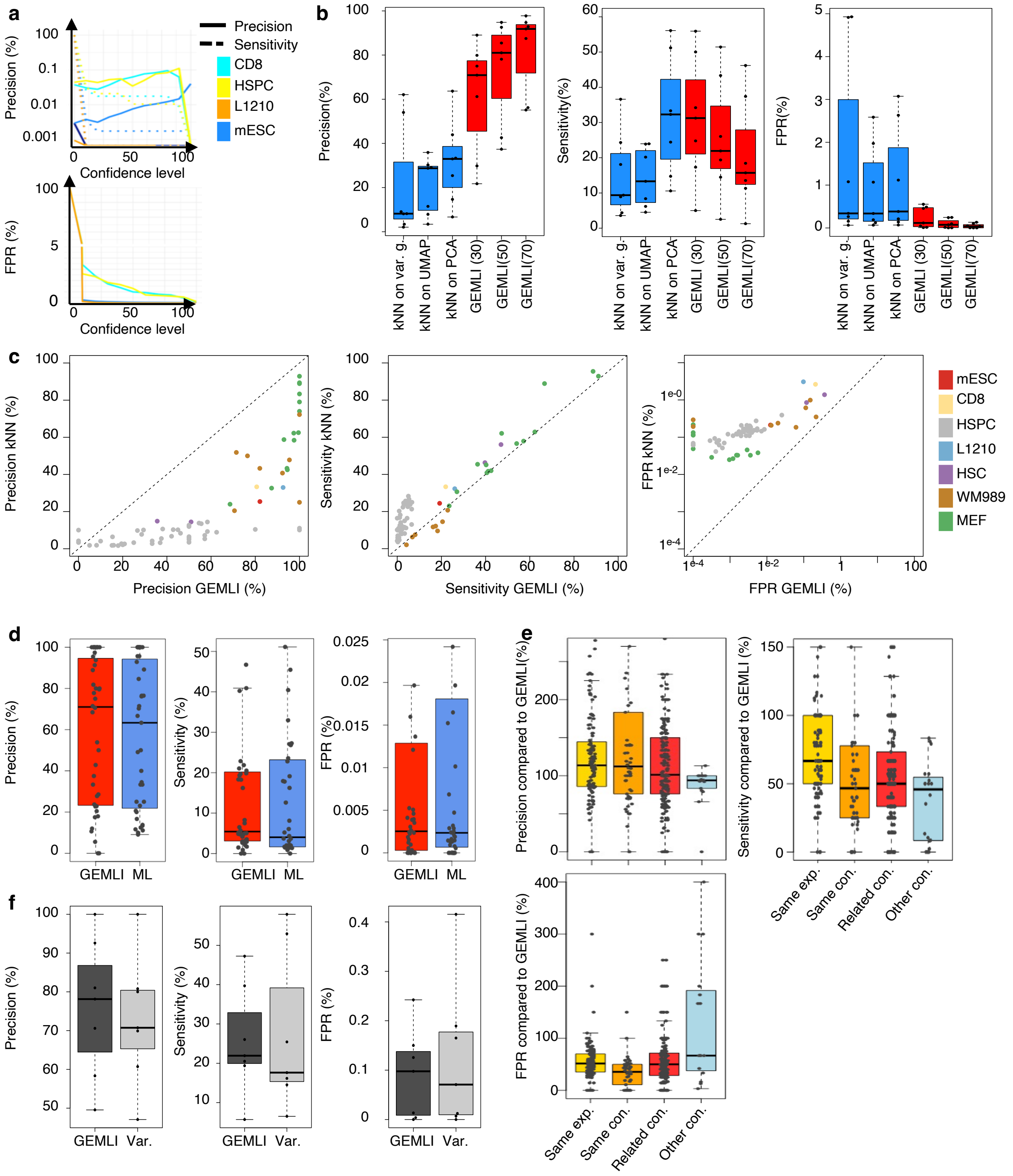

fig. S9: Comparison of GEMLI performance to other lineage assignments. (a) Precision-sensitivity and FPR curves of GEMLI predictions of random cell lineages for the indicated cell types (1 dataset for each). (**b**) Comparison of precision (left), sensitivity (middle) and FPR (right) for kNN clustering with k=2 based on variable genes (var. g.), UMAP, or PCA and GEMLI predictions at three confidence levels (30, 50, and 70). The mean over datasets is shown (n=1 mESC, CD8, L1210, n=2 HSC, n=8 WM989, n=14 MEF, n=44 HSPC). (**c**) Precision for kNN clustering with k=2 based on PCA and GEMLI at confidence level 50 for individual datasets as in (b) colored by cell type. (**d**) Precision (left), sensitivity (middle) and FPR (right) at confidence level 50 for GEMLI predictions using GEMLIs gene selection or a machine-learning (ML) based gene set for 40 datasets of several cell types (n=1 for mESCs, CD8, L1210, n=2 for HSC, n=6 for MEF, n=8 for WM989, n=21 for HSPC). (**e**) Percentage of GEMLI lineage prediction precision (top left), sensitivity (top right), and FPR (bottom left)) gained by using memory gene sets called on datasets from the same experiment (same exp.), same cell culture condition with same starting cell type (same con.), a same culture condition with a related starting cell type (related con.), or a greatly different cell culture condition with unrelated starting cell type (other con.). Datasets of differentiating

HSPC were used for comparisons across same experiment, same condition, and related conditions (datasets n =20 across 4 experiments and 3 conditions (LK condition datasets n=3, LSK condition datasets n=3 and 9 respectively, LK_LSK condition datasets n= 5). For the comparison across other conditions (unrelated starting cell types), datasets of LK cells, CD8, L1210, MEF and mESC were compared (n=1 each). (**f**) Precision, sensitivity and FPR for GEMLI predictions using the GEMLI gene set (dark grey) or Seurat variable genes (light grey; var.) at confidence level 50 for one dataset in seven cell types (mESC, CD8, L1210, WM989, HSC, HSPC, MEF). Boxplots as in fig. S2. Source data are provided as a Source Data file.

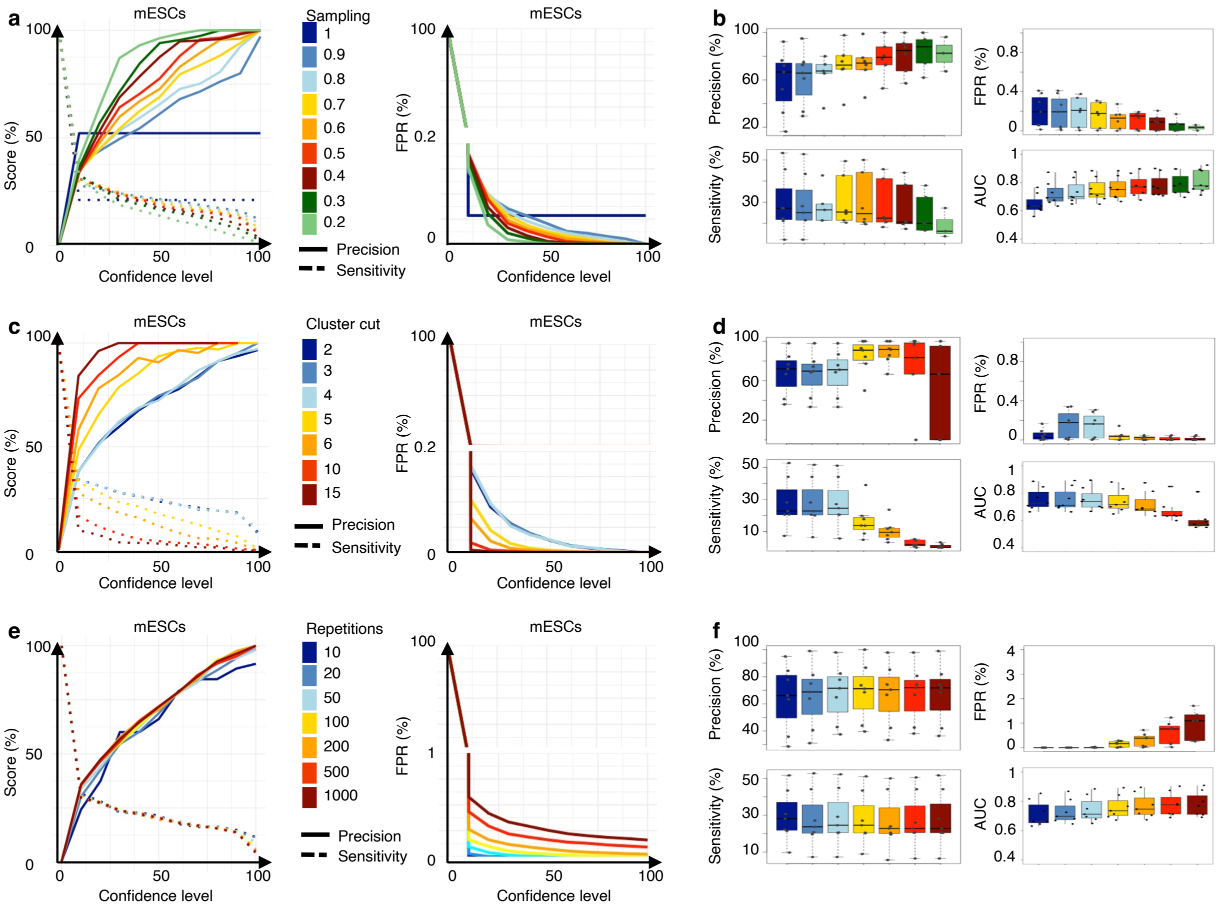

fig. S10: Influence of GEMLI parameters on lineage predictions. (**a**) Precision-sensitivity (left) and FPR (right) curves of lineage predictions in the mESC dataset (n=1) sampling different fractions of genes during each iterative clustering as indicated. (**b**) Precision (top left), sensitivity (bottom left), FPR (top right), and AUC (bottom right) for mESC, CD8, L1210, HSPC, HSC, WM989, and MEF datasets (n=1 each) at a confidence level of 50 using different fractions of genes (color-coded) during each iterative clustering as in (a). (**c**) Precision-sensitivity (left) and FPR (right) curves of lineage predictions in the mESC dataset splitting clusters into the number indicated (colors) during each clustering iteration round. (**d**) Precision, sensitivity, FPR, and AUC as in (b) at a confidence level of 50 for datasets as in (b) splitting clusters as in (c; color-coded). (**e**) Precision-sensitivity (left) and FPR (right) curves of lineage predictions in the mESC dataset repeating the iterative clustering different number of times as indicated. (**f**) Precision, sensitivity, FPR and AUC as in (b) at a confidence level of 50 for datasets as in (b) using different numbers of repetitions (color-coded) as in (e). Boxplots as in fig. S2. Source data are provided as a Source Data file.

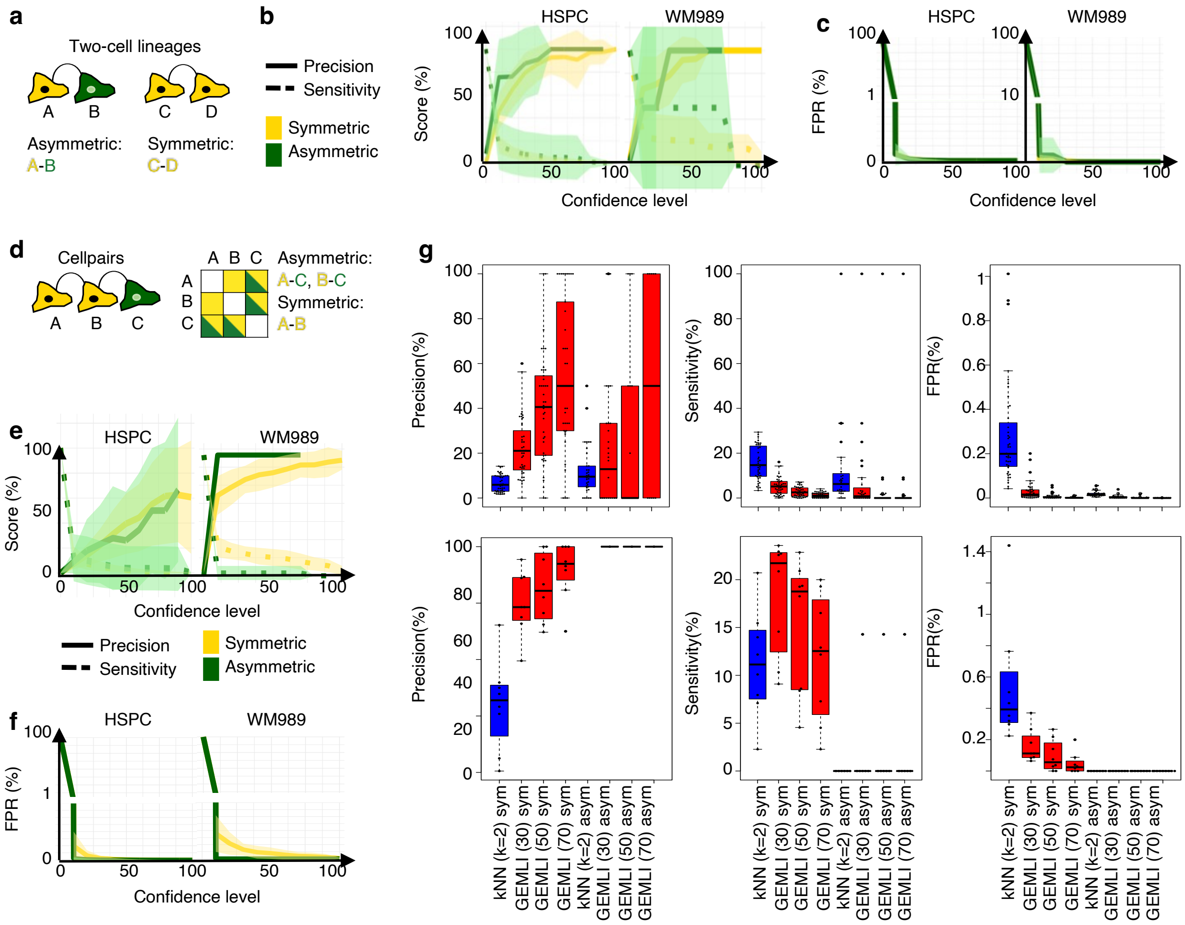

fig. S11: GEMLI predictions of symmetric and asymmetric lineages. (**a**) Scheme of symmetric (composed of one cell type) and asymmetric (composed of two cell types) classification of two-cell lineages. (**b-c**) Precision-sensitivity (b) and FPR (c) curve for symmetric and asymmetric two-cell lineages in the HSPC (n=44) and WM989 datasets (n=8; mean=line; shade=S.D.). (**d**) Scheme of symmetric and asymmetric classification of cell pairs within lineages. (**e-f**) Precision-sensitivity (e) and FPR (f) curve for symmetric and asymmetric cell pairs in the HSPC and WM989 datasets as in (b-c). (**g**) Precision (left), sensitivity (middle) and FPR (right) for cell pairs within symmetric (sym) and asymmetric (asym) lineages from GEMLI predictions (confidence value 30, 50 or 70) and clusters based on kNN (k=2) in the HSPC datasets (top; n=44) and WM989 datasets (bottom; n=8). Source data are provided as a Source Data file.

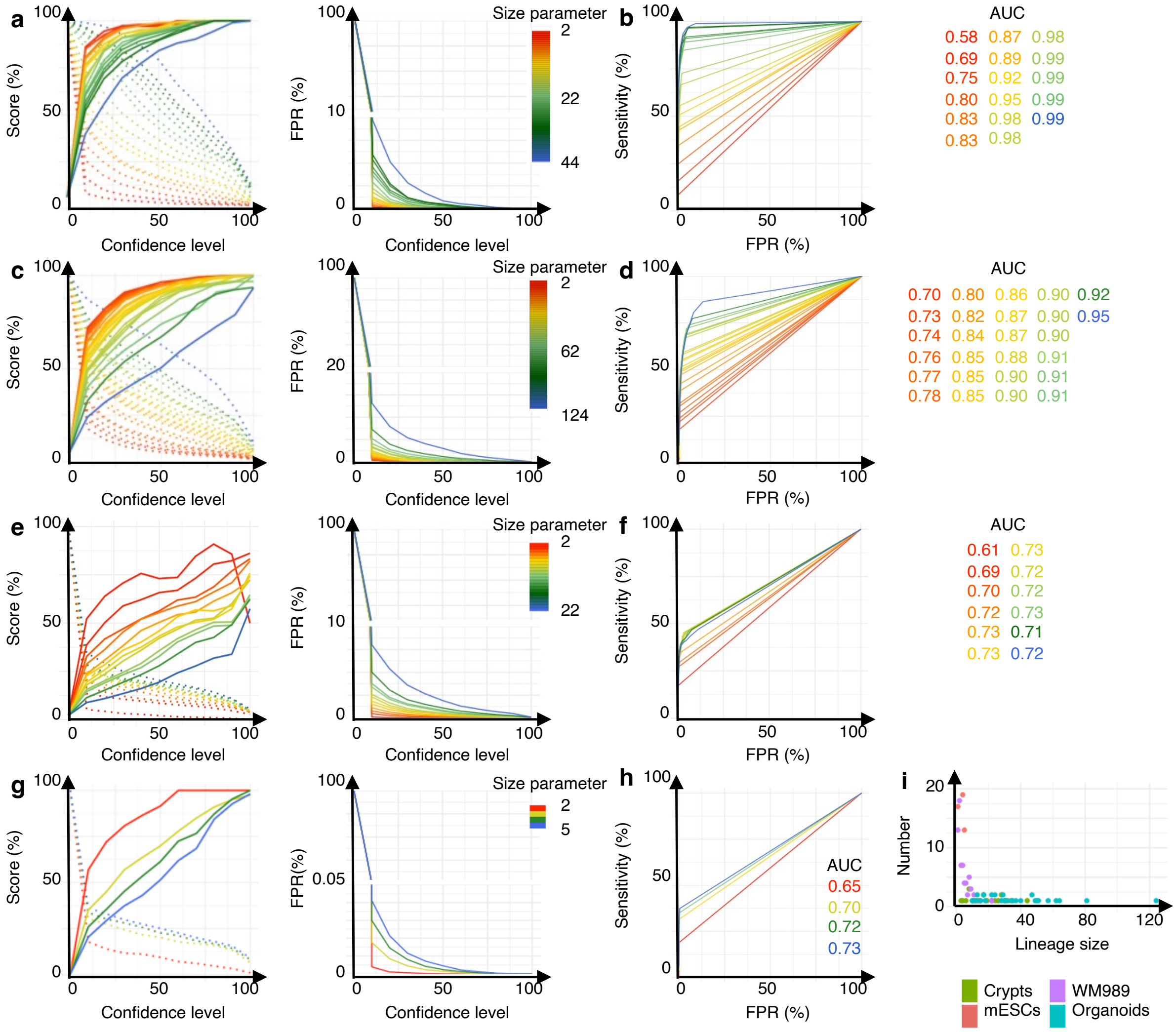

fig. S12: Influence of lineage size parameters on GEMLI lineage predictions. (**a**) Precision-sensitivity curves (left; precision: line, sensitivity: dotted line) and FPR (right) curves in the crypt dataset (n=1) for GEMLI runs with different lineage size parameters (color) within the ground truth lineage size range as indicated. (**b**) ROC curves and AUC values for predictions as in (a). (**c-d**) Analysis as in (a-b) for the organoid dataset (n=1). (**e-f**) Analysis as in (a-b) for one WM989 dataset. (**g-h**) Analysis as in (a-b) for the mESC dataset (n=1). (**i**) Number of lineage sizes in the ground truth lineage annotation for datasets as in (a-h). Source data are provided as a Source Data file.

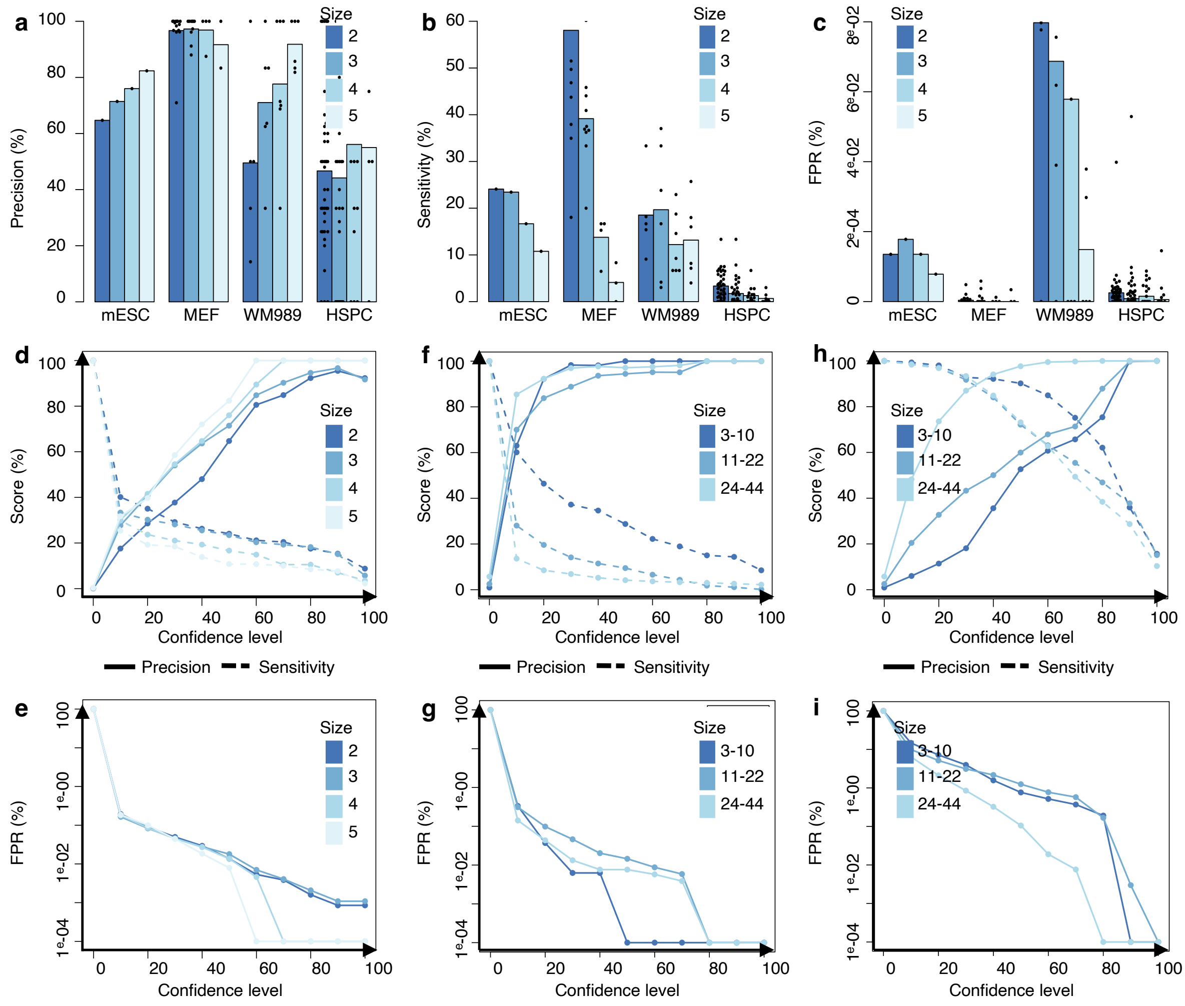

fig. S13: Performance of GEMLI for different lineage sizes. (a) Precision of GEMLI predictions for different ground truth lineage sizes at confidence level 50 in the mESC, MEF, WM989, and HSPC datasets. (**b-c)** Same representation as in (a) for sensitivity (b) and FPR (c). (**d)** Precision-sensitivity curves for different ground truth lineage sizes as indicated in mESCs. (**e)** FPR curve for different ground truth lineage sizes as indicated in the mESCs dataset. (**f)** Precision-sensitivity curves for GEMLI predictions with a lineage size parameter of 5 on the crypts dataset for three ground truth lineage size bins as indicated. (**g)** FPR curve for GEMLI predictions with a lineage size parameter of 2-5 on the crypts dataset for three ground truth lineage size bins as indicated. (**h-i)** Same representation as in (f-g) for GEMLI predictions with a lineage size parameter of 2-40. For a-c: shown is the median over (n=datasets; ESC n=1, MEF n=14, WM n=8, HSPC n=44). Source data are provided as a Source Data file.

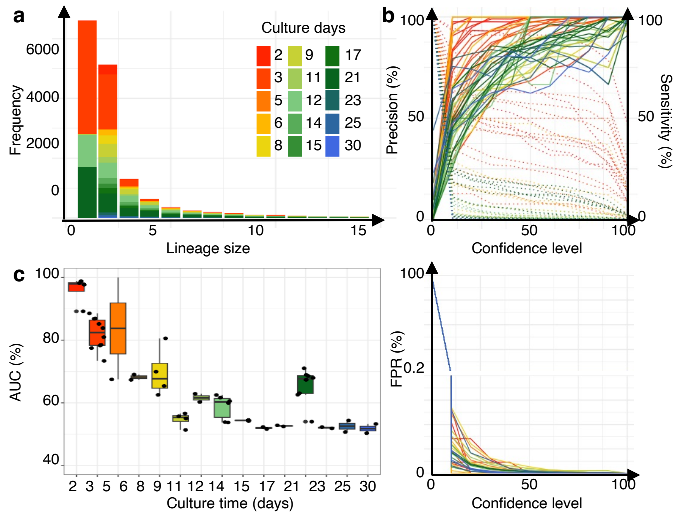

fig. S14: Performance of GEMLI over extended time spans. (**a**) Frequency of lineage sizes in the ground truth lineage annotation for timepoints of up to 30 days during a MEF reprogramming time course experiment (n=4,10,2,2,4,4,2,6,2,2,2,8,2,2,2 datasets, respectively, for indicated days). (**b**) Precision-sensitivity (top; line=precision, dotted line=sensitivity) and FPR (bottom) curves of lineage predictions in the MEF datasets as in (a; also color-coding). (**c**) AUC values for lineage predictions across the MEF reprogramming time course as in (a). Boxplots as in fig. S2. Source data are provided as a Source Data file.

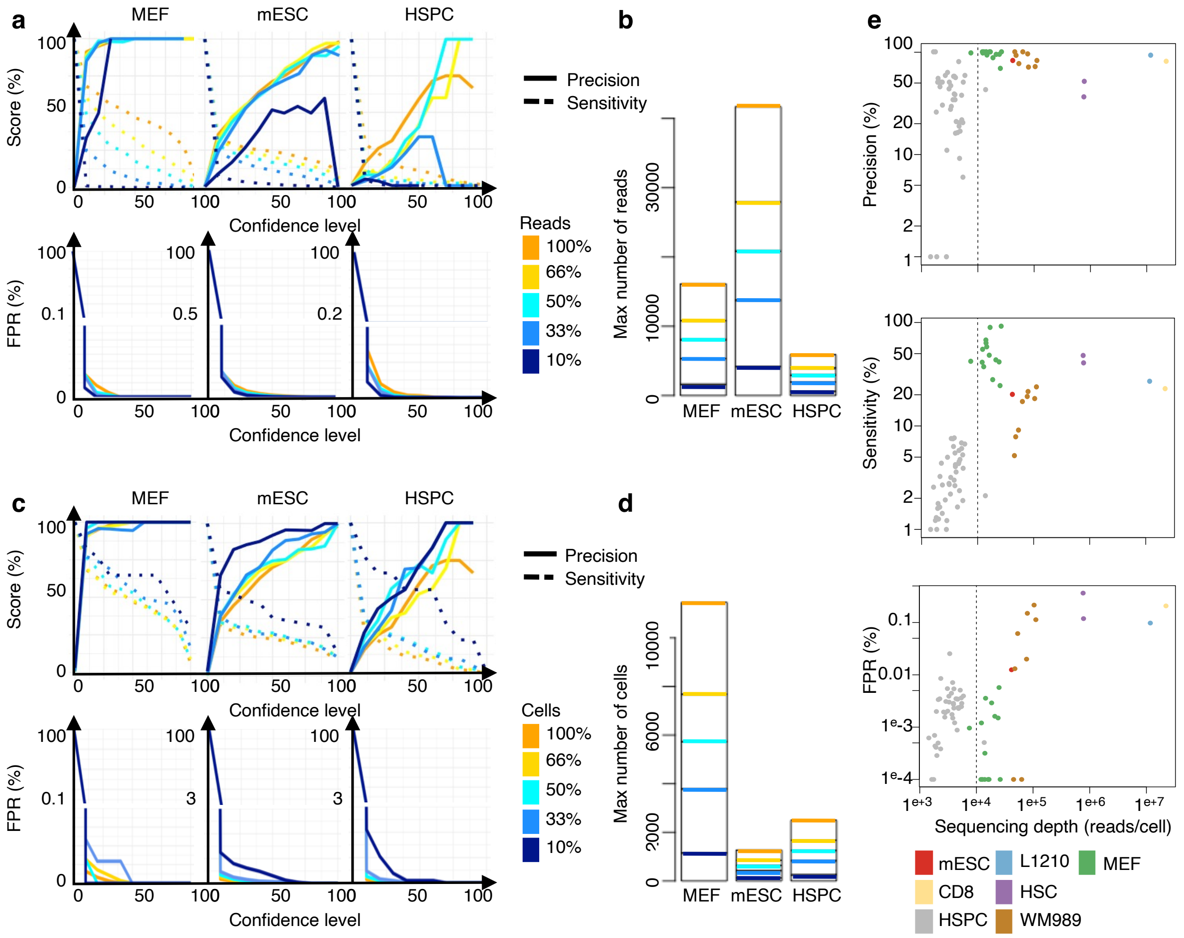

fig. S15: Relation of dataset read and cell numbers on GEMLI performance. (**a**) Precision-sensitivity (top) and FPR (bottom) curves for one MEF, mESC, and HSPC dataset after subsampling reads as indicated. (**b**) Read number in the indicated dataset and subsampling category as in (a). (**c**) Same representation as in (a) for subsampling of cells. (**d**) Cell number in the indicated dataset and subsampling category as in (c). (**e**) Precision (top), sensitivity (middle) and FPR (bottom) with respect to sequencing depth of datasets in 7 different cell types (color coded; n=datasets; n=1 for mESC, CD8, L1210, n=8 for WM989, n=2 for HSC, n=14 for MEF, n=44 for HSPC). A confidence value of 50 was used. Source data are provided as a Source Data file.

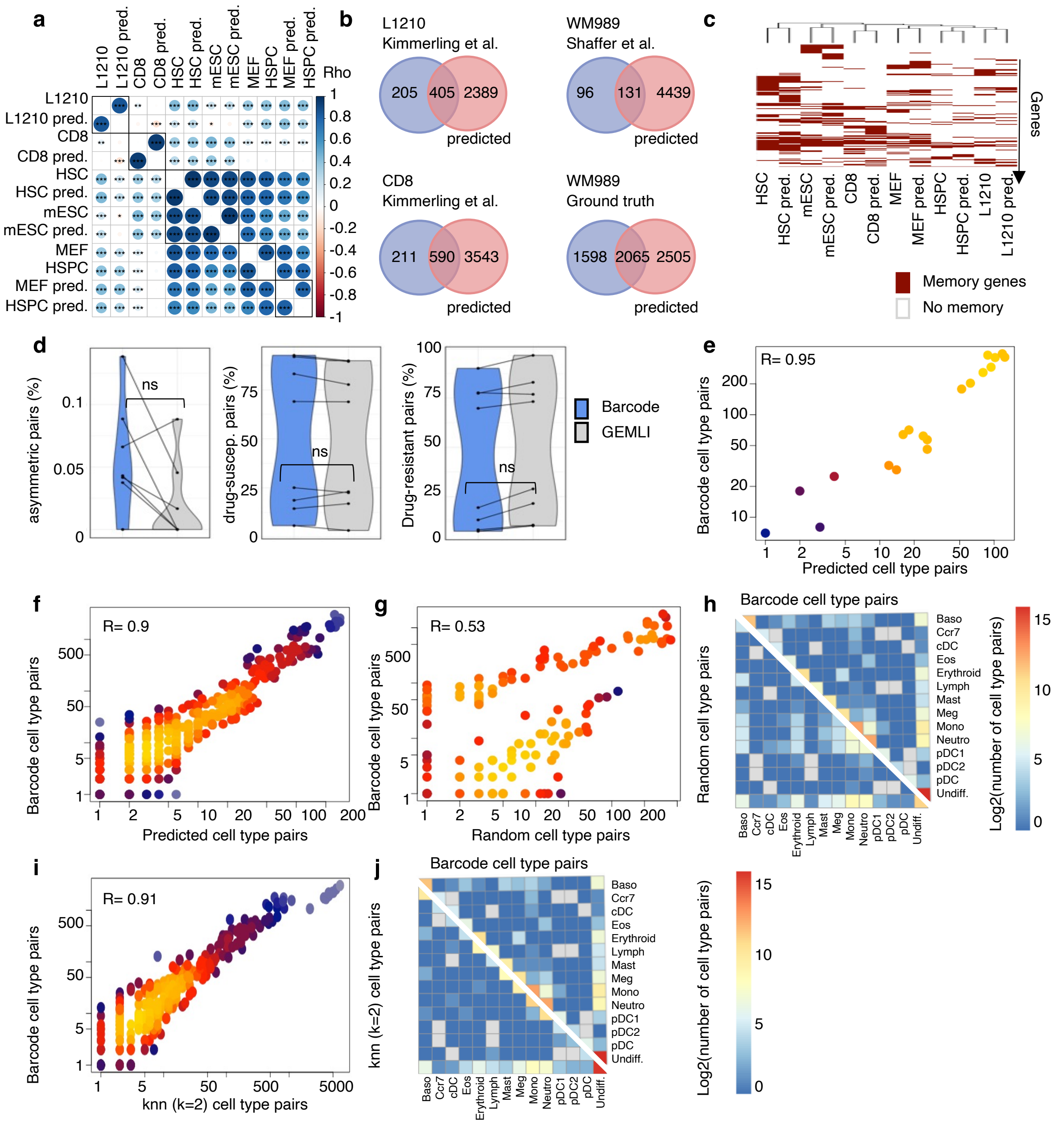

fig. S16: GEMLI lineage predictions can identify memory genes and cell fate decisions. (**a**) Spearman rank correlation matrix of GO-term enrichment (top 500) in memory genes of ground truth or predicted (pred.) cell lineages at confidence level 30 in the indicated datasets (n=1; Spearman ranks test: ∗p-value<0.05; ∗∗p-value<0.01; ∗∗∗p-value<0.001). (**b**) Overlap of memory genes of CD8, L1210 and WM989 cells from Kimmerling et al. 2016 and Shaffer et al. 2020 with the memory genes called on predicted lineages in these cell types (one dataset each as in (a)). (**c**) The overlap of memory genes called on ground truth cell lineages, and memory genes called on predicted (pred.) lineages across cell types (n=1 dataset each). For the human WM989 cells overlap is in a Venn diagram (left). (**d**) Percentage of asymmetric (left), entirely drug-susceptible (middle), and entirely drug-resistant (right) cell pairs in barcode and GEMLI lineages across WM989 datasets (n=8). Density plot and individual paired datapoints are represented. (**e**) The number of cell pairs in barcode and predicted lineages in all possible cell type pair categories across WM989 datasets as in (d). Each dot represents one cell type combination in one dataset. Coloring represents density in the scatter plot. Spearman rank correlation is given. (**f**) Scatterplot as in (e) showing the number of cell pairs in barcode and predicted lineages in all cell type combinations categories across all HSPC datasets (n=44). (**g**) Scatterplot as in (f) for random cell pairs. (**h**) Heatmap of the number (sum) of cell pairs in barcode (top right) and random cell lineages (bottom left) in all possible cell type pair categories in the HSPC datasets as in (f). (**i**) Representation as in (f) for barcode cell pairs

and cells grouped by kNN clustering (k=2, based on PCA). (**j**) Representation as in (h) for barcode cell pairs and cells grouped by kNN clustering as in (i). For (d) significance was tested using a two-sided Mann-Whitney U test ($p = 0.05$). Confidence level of predictions in (b-j) is 50. For abbreviations in panels h and j, see Methods. Source data are provided as a Source Data file.

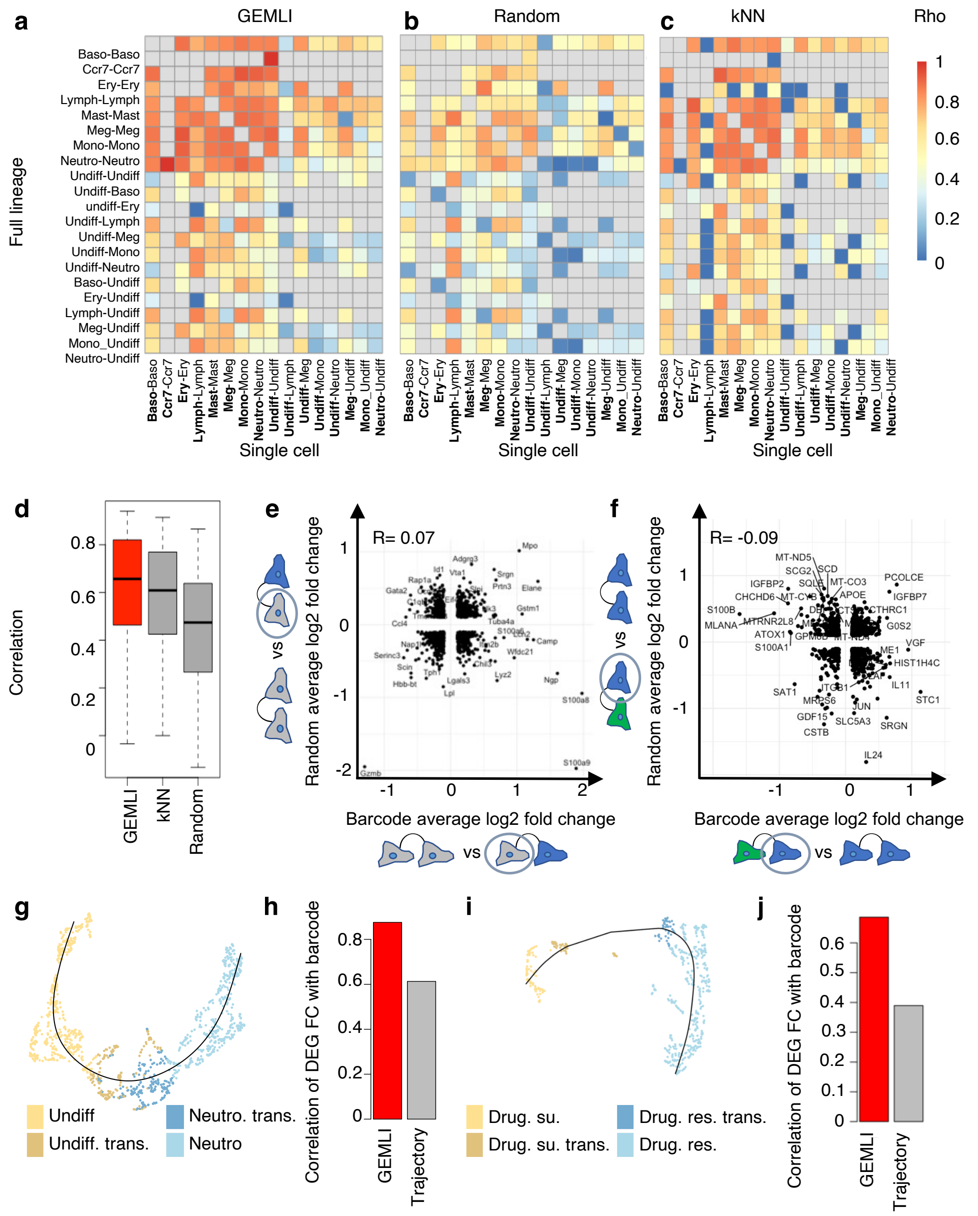

fig. S17: GEMLI lineage predictions can identify gene expression in specific lineage types. (**a**) Mean Spearman rank correlation of DEG called between cell pairs in the indicated lineages for barcode and GEMLI lineages in the HSPC datasets (n=44). Full lineages (y-axis) were compared to single members of other lineages (x-axis). For the single cells, the bold cell of the lineage was used for DEG calling. Categories for which no DEG could be called are gray or omitted. For abbreviated cell type see Methods. (**b**) Same representation as in (a) for predicted lineages after exchanging cells against random cells of the same cell type. (**c**) Same representation as in a for cell pairs clustered by kNN (k=2). (**d**) Mean Spearman rank correlation for enrichment of DEG called on predicted, random lineages, or kNN clustered cells and barcode ground truth as in (a-c) in the HSPC datasets (n=44). (**e**) Average log2 fold change for enrichment of DEG called in the HSPC datasets (n=44) between barcode symmetric undifferentiated and entire asymmetric cell lineages (undifferentiated-neutrophil), against DEG called on predicted lineages with cells exchanged by random cells of the same cell type (grey: undifferentiated; blue: neutrophil). Dots: DEGs. Spearman rank correlation is given. The 20 highest and 10 lowest enriched genes are named. (**f**) Comparison as in (e) for symmetric and asymmetric lineages in the WM989 datasets (n=8 summed; blue: drug-susceptible, green: primed for drug-resistance). (**g**) Trajectory analysis of undifferentiated cells (Undiff) and neutrophils (Neutro) in one dataset. Cells close to the transition area according to pseudo-time are highlighted. (**h**) Correlation of fold-changes (FC) in gene expression of undifferentiated cells far or close to the

transition. GEMLI: Correlation of DEG FC based on barcodes and predictions. Trajectory: Correlation of DEG FC based on barcodes and cells selected based on trajectory analysis as in (g). (**i**) Trajectory as in (g) for drug susceptible (Drug su.) and drug resistant (Drug res.) WM989 cells in one dataset. (**j**) Correlation of FC as in (h) based on barcode lineages and trajectory analysis as shown in (i) respectively for WM989 datasets (n=8). Source data are provided as a Source Data file.

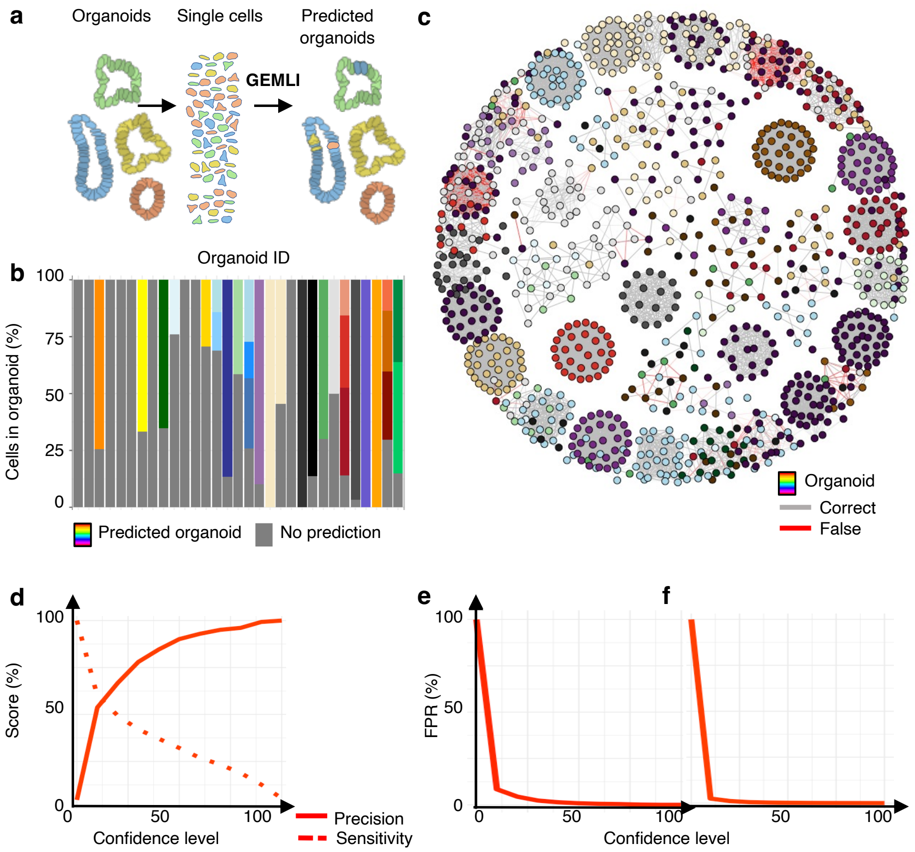

fig. S18: GEMLI lineage predictions correctly assign cells to individual organoids and crypts. (**a**) Scheme of GEMLI organoid predictions. (**b**) Percentage of cells predicted as individual lineages (sizes 8-40 represented) for each organoid at confidence level 50. (**c**) Predictions of organoids at confidence level 70. (**d**) Precision-sensitivity curve of lineage predictions in the organoid dataset. (**e**) FPR curves of lineage predictions in the organoid dataset. (**f**) Same representation as in (e) for the crypt dataset. Size parameter for all predictions is 2-40. Source data are provided as a Source Data file.

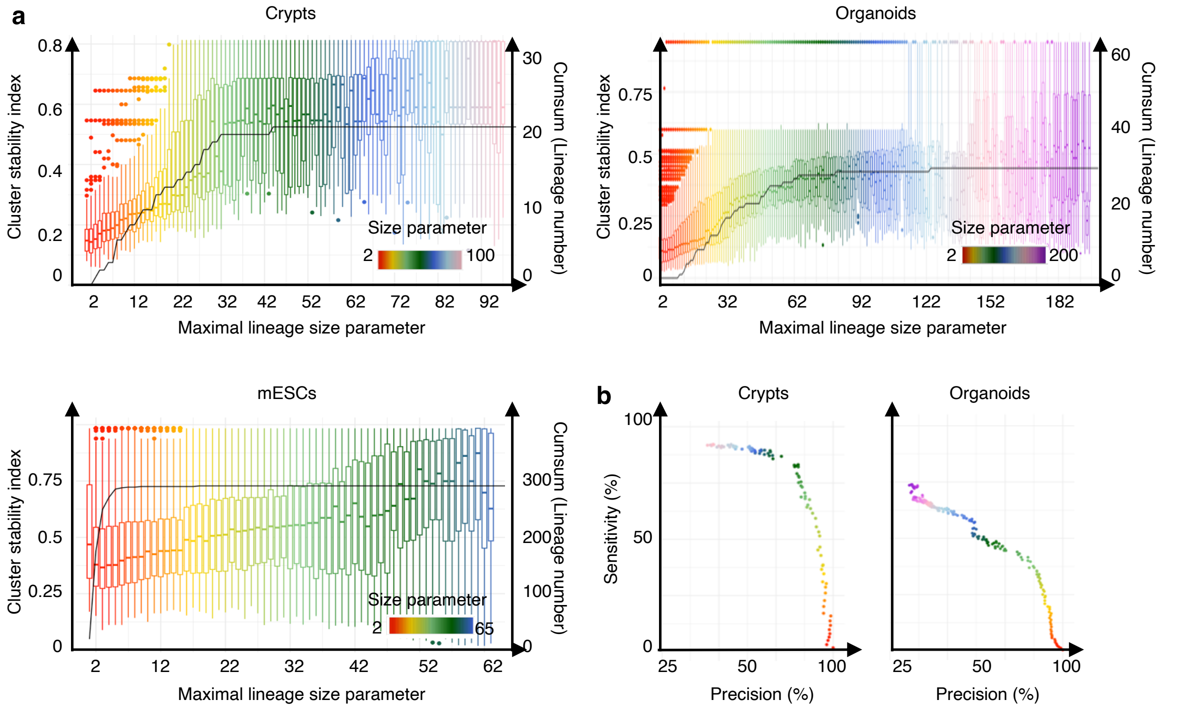

fig. S19: Lineage size structure in scRNA-seq datasets can be estimated using stability of lineage predictions. (**a**) Relation of cluster stability of predicted lineages with increasing maximal lineage size parameters (color and x-axis) and its relation to ground truth lineage sizes (Cumsum: cumulative sum; black lines) in two datasets of multicellular structures (top; crypt and organoid dataset; n=1 each) and one dataset of cells in culture (bottom; mESCs; n=1). Boxplots as in fig. S2. (**b**) Relation of sensitivity and precision of lineage predictions for different maximal lineage size parameters as in (a) for crypt and organoid dataset at confidence level 50. Source data are provided as a Source Data file.

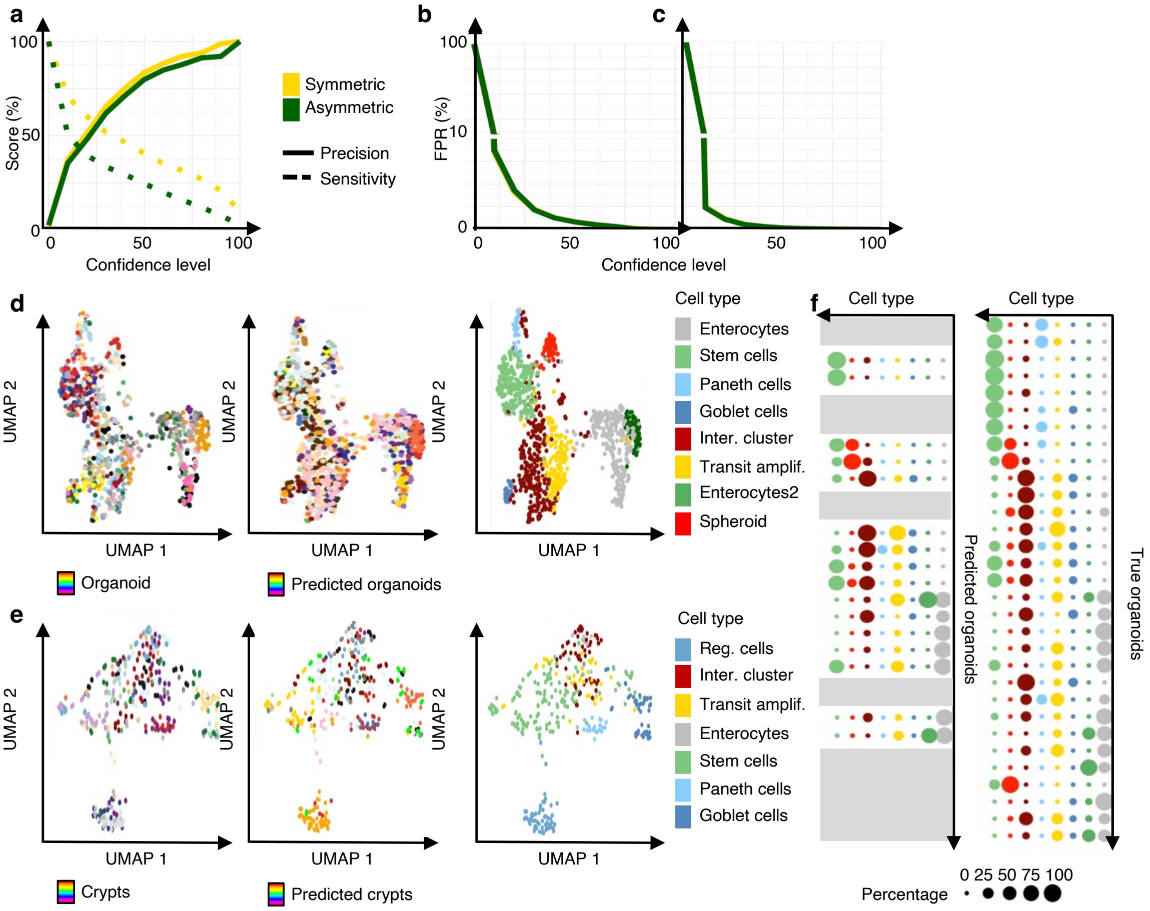

fig. S20: GEMLI predicts the cell type composition of individual organoids and crypts. (**a**) Precision-sensitivity curve for lineage predictions of lineage cell pairs with one (symmetric) or two cell types (asymmetric) in the organoid dataset. (**b**) FPR curve for lineage predictions of symmetric and asymmetric lineage cell pairs as in (a) in the organoid dataset. (**c**) Same representation as in (b) for the crypt dataset. (**d**) UMAP of the organoid dataset colored by organoid (left), predicted organoid (middle) and cell type (right). (**e**) Same representation as in (d) for the crypt dataset. (**f**) The percentage of cells belonging to the indicated cell type (coloring as in (d)) for individual organoids (right) and for predicted lineages of 5-40 cells (left) aligned to the ground truth organoid the majority of cells belongs to. Confidence level is 50. Inter: intermediate, amplif: amplifying, reg: regenerative. Size parameter for all predictions is 2-40. Source data are provided as a Source Data file.

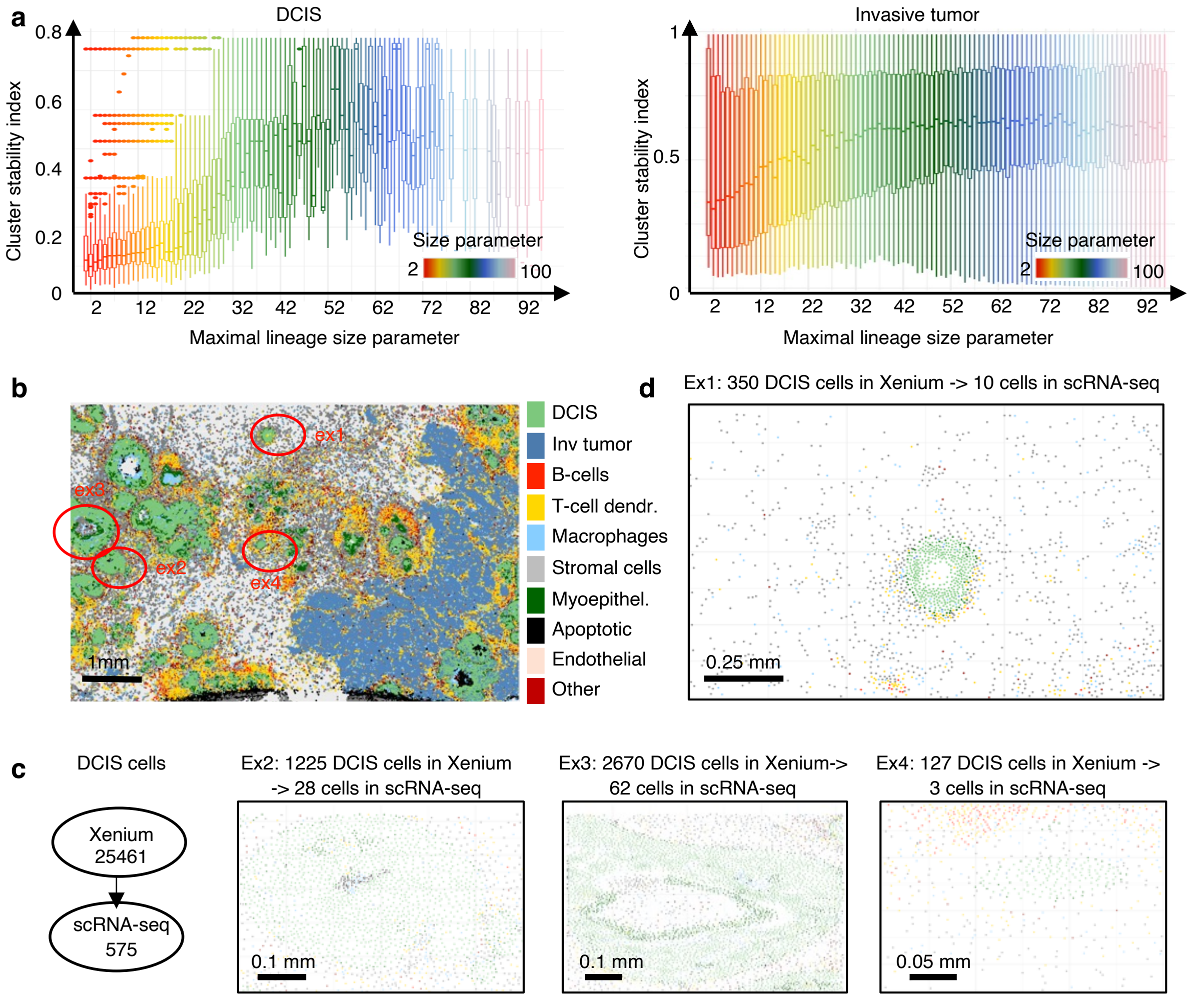

fig. S21: Stability of GEMLI predictions informs on nodule structure of breast DCIS cells. (a) Relation of sc3 cluster stability of predicted lineages with increasing maximal lineage size parameter (color and x-axis) for DCIS (left) and invasive tumor (right) cells in the breast cancer scRNA-seq dataset. Boxplot as in fig. S2. (**b**) Xenium in situ sequencing map for all cells in the breast cancer dataset colored by cell type. Four DCIS nodules are highlighted in red. Inv tumor: invasive tumor, T-cell dendr: T-cell dendritic cell. (**c**) Number of DCIS cells in the Xenium and scRNA-seq datasets. (**d**) Zoomed in view for the four DCIS nodules highlighted in (b). Number of DCIS cells in the Xenium data and estimated number of corresponding cells in the scRNA-seq breast cancer dataset. Recovery of cells in the scRNA-seq dataset (2,3%) was estimated based on the total number of DCIS cells in the Xenium and scRNA-seq data as in (c). Source data are provided as a Source Data file.

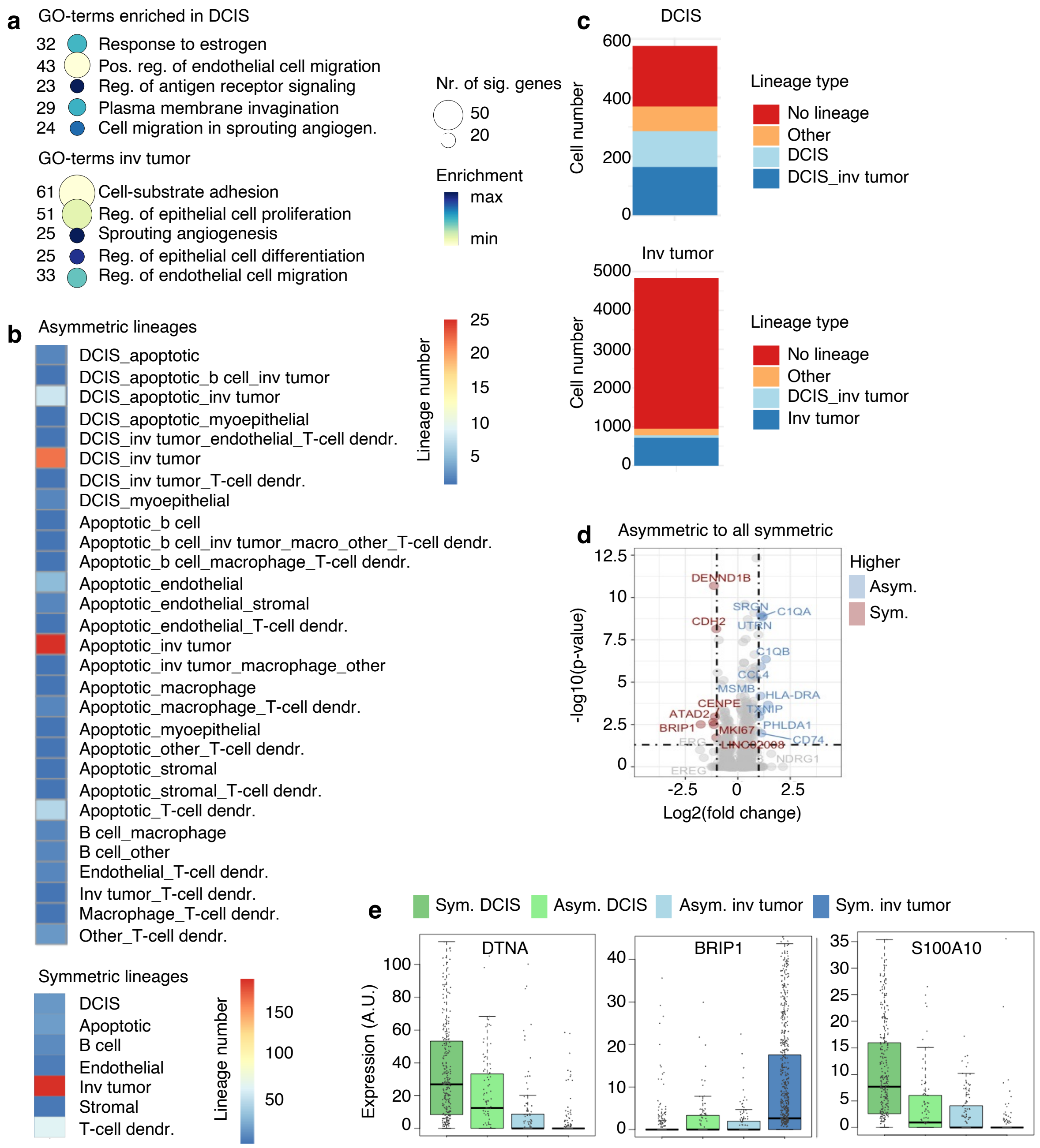

fig. S22: GEMLI identifies memory genes and gene expression specific to lineages at the transition from DCIS to invasive breast cancer (a) Selected GO-terms enriched in memory genes called using predicted DCIS and invasive tumor lineages in the breast cancer scRNA-seq dataset. The number of significant (sig.) genes is indicated. (**b**) Number of predicted lineages with members in several cell types (asymmetric lineages) or in one cell type (symmetric lineages) across all cell types in the breast cancer scRNA-seq dataset. (**c**) Number of DCIS (top) and invasive tumor (bottom) cells with respect to their predicted lineage type. “Other” refers here to any other cell type than DCIS and invasive tumor. (**d**) Volcano plot for DEG called between all cells part of asymmetric (asym.) lineages and all cells part of symmetric (sym.) lineages as indicated. The top 9 highest and lowest enriched DEG are named. (**e**) Expression of selected DEG across individual DCIS and invasive tumor cells being part of symmetric and asymmetric lineages as in (d). All predictions are at confidence level 50, with lineage size parameter 2-20. Boxplot as in fig. S2. Inv tumor: invasive tumor, T-cell dendr: T-cell dendritic cell, Macro: macrophage, Reg.: regulation, Pos.: positive. Source data are provided as a Source Data file.

Supplementary Tables:

table S1: Comparison of the GEMLI algorithm with classical single-cell clustering approaches

	Classic clustering	GEMLI
**Gene selection**	Highly variable genes across expression ranges, empirically enriched for cell type / state markers	Very highly abundant genes (independent of their variability) and variable genes that are highly abundant, empirically enriched for memory
**Dimensionality reduction**	Commonly a PCA, followed by UMAP or tSNE	No dimensionality reduction, all genes are considered independently
**Distance**	Based on dimensionality reduction	Based on correlation of expression ranks of memory enriched genes
**Cluster definition**	Single clustering step based on overall similarity in dimensionality reduction or gene expression network (e.g. k-means or kNN)	Iterative clustering to a specific cluster size, repeated with varying subsets of memory enriched genes, consensus informs clusters
**Cluster identity**	Clusters commonly represent cell types or cell states, independently of cell lineage.	Clusters represent cell lineages, independently of cell type and cell state.
**Cluster sizes**	Vastly varying in size (from 10s to 1000s) depending on the cell types present in the data.	Clusters are not of a single size but more homogeneous. They commonly recapitulate the lineage sizes present in the data.

table S2: Filtering thresholds for the MEF datasets

Dataset	Mitochondrial read (%)	Reads
D0_2	1-14	2000
D0	1,2-7,5	7000
D3_2	3-14	6000
D3	2-10	6000
D6_2	3-14	6000
D6	3-20	4000
D9_2	2-18	6000
D9	2-18	6000
D12_2	2-18	4000
D12	2-18	3000
D15_2	2-14	2000
D15	2-18	4000

D21_2	2-20	2000
D21	2-18	4000
D28_2	3-18	2500
D28	3-18	3000
