# Supplementary material for: Gene-expression memory-based prediction of cell lineages from scRNA-seq datasets
Source: Nat Commun. 2024 Mar 29;15:2744. doi: 10.1038/s41467-024-47158-y (PMC10980719; doi:10.1038/s41467-024-47158-y)
Supplement: Supplementary file 3 — Description of Additional Supplementary Files [file 41467_2024_47158_MOESM3_ESM.pdf]

Description of Additional Supplementary Files:

Supplementary Data 1: Overview of all lineage-annotated scRNA-seq datasets used in the study. Sheet 1: Overview of all lineage-annotated scRNA-seq datasets of invitro cultured cells used in the study including source publication, the state of cells, sequencing platform, culture time, and lineages sizes. Sheet 2: Overview of dataset used in each figure of the paper. Sheet 3: Overview of all in-vivo derived lineageannotated scRNA-seq datasets of in-vivo derived cells used in the study including the source publication, the state of cells, and sequencing platform.

Supplementary Data 2: List of memory genes called based on a high coefficient of variation squared between lineage means in different cell types. Memory genes called on the mESC dataset (sheet 1), one MEF dataset as indicated (sheet 2), the CD8 dataset. (sheet 3), the L1210 dataset (sheet 4), one HSPC dataset as indicated (sheet 5), one WM989 dataset as indicated (sheet 6), one HSC dataset as indicated (sheet 7). In each sheet, every gene of the dataset is given a p-value generated by comparison of the true lineage value with 20 repeated random samplings. This indicates the likelihood to be categorized as memory gene. Further the genes categorization as qualitative or quantitative gene is indicated.

Supplementary Data 3: GO-terms enriched in quantitative and qualitative memory genes in different cell types. GOterm enrichment in quantitative and qualitative memory genes of the mESC dataset (sheet 1 & 6 respectively), the CD8 dataset (sheet 2 & 7), the BIDDY_D0_2 MEF dataset (sheet 3 & 8), the LK_D2_exp1_library_d2_2 HSPC dataset (sheet 4 & 9), the L1210 dataset (sheet 5 & 10). Indicated in each list are GO.ID, associated GO-term, the number of annotated genes (annotated), the number of significantly enriched genes (significant), the number of expected significantly enriched genes (expected), the p-value of the classic Fisher test, and the enrichment value as generated by the TopGO R package (see Methods).

Supplementary Data 4: GO-terms enriched in all memory genes called using ground truth and predicted lineage information across cell types. GO-term enrichment in memory genes called based on the coefficient of variation squared in lineage means in different cell types using lineages of ground truth (GT) or GEMLI predictions (pred) at confidence level 30. Datasets for which data is given are the mESC dataset (sheet 1 GT, sheet 7 pred), the CD8 dataset (sheet 2 GT, sheet 8 pred), the L1210 dataset (sheet 3 GT, sheet 9 pred), one MEF dataset (BIDDY_D0_2, sheet 4 GT, sheet 10 pred), one HSPC dataset (LK_D2_exp1_library_d2_2, sheet 5 GT, sheet 11 pred), one HSC dataset (Wehling_1, sheet 6 GT, sheet 12 pred). Indicated in each list are GO.ID, associated GO-term, the number of annotated genes (annotated), the number of significantly enriched genes (significant), the number of expected significantly enriched genes (expected), the pvalue of the classic Fisher test, and the enrichment value as generated by the TopGO R package (see Methods).

Supplementary Data 5: GO-terms enriched in all memory genes called on predicted human breast cancer DCIS and invasive tumor lineages. GO-terms enriched in lineages predicted in DCIS cells (sheet 1), and invasive tumor cells (sheet 2). Indicated in each list are GO.ID, associated GO-term, the

number of annotated genes (annotated), the number of significantly enriched genes (significant), the number of expected significantly enriched genes (expected), the pvalue of the classic Fisher test, and the enrichment value as generated by the TopGO R package. Further the number of significantly enriched genes in 100 expression matched controls is given. Percentages 50%, 75%, 90%, 95%, 99% and 100% show quantiles of the occurrence of a given GOterm across random sets (e.g. 23 at 95% means that 95 out of the 100 random samples have 23 or fewer gene in this GO-term, see Methods).

Supplementary Data 6: Hyperparameters for the neural network to optimized gene selection for lineage predictions based on variability and mean expression of genes.
