# Supplementary material for: Gene-expression memory-based prediction of cell lineages from scRNA-seq datasets
Source: Nat Commun. 2024 Mar 29;15:2744. doi: 10.1038/s41467-024-47158-y (PMC10980719; doi:10.1038/s41467-024-47158-y)
Supplement: Supplementary file 10 — Reporting Summary [file 41467_2024_47158_MOESM10_ESM.pdf]

nature portfolio

Corresponding author(s): Almut S Eisele and David M Suter

Last updated by author(s): 10.02.2024

Reporting Summary

Nature Portfolio wishes to improve the reproducibility of the work that we publish. This form provides structure for consistency and transparency in reporting. For further information on Nature Portfolio policies, see our Editorial Policies and the Editorial Policy Checklist.

Statistics

For all statistical analyses, confirm that the following items are present in the figure legend, table legend, main text, or Methods section.

n/a	Confirmed	
☐	☒	The exact sample size (*n*) for each experimental group/condition, given as a discrete number and unit of measurement
☐	☒	A statement on whether measurements were taken from distinct samples or whether the same sample was measured repeatedly
☐	☒	The statistical test(s) used AND whether they are one- or two-sided<br>*Only common tests should be described solely by name; describe more complex techniques in the Methods section.*
☒	☐	A description of all covariates tested
☒	☐	A description of any assumptions or corrections, such as tests of normality and adjustment for multiple comparisons
☐	☒	A full description of the statistical parameters including central tendency (e.g. means) or other basic estimates (e.g. regression coefficient) AND variation (e.g. standard deviation) or associated estimates of uncertainty (e.g. confidence intervals)
☐	☒	For null hypothesis testing, the test statistic (e.g. *F*, *t*, *r*) with confidence intervals, effect sizes, degrees of freedom and *P* value noted<br>*Give P values as exact values whenever suitable.*
☒	☐	For Bayesian analysis, information on the choice of priors and Markov chain Monte Carlo settings
☒	☐	For hierarchical and complex designs, identification of the appropriate level for tests and full reporting of outcomes
☐	☒	Estimates of effect sizes (e.g. Cohen's *d*, Pearson's *r*), indicating how they were calculated

Our web collection on statistics for biologists contains articles on many of the points above.

Software and code

Policy information about availability of computer code

Data collection	During data collection we used the software FACSDiva (BD; flow cytometry) and IN Cell ANalyzer 2200 software (GE Healthcare; microscopy).
Data analysis	The R package described in the paper is available at https://github.com/UPSUTER/GEMLI.<br><br>For barcode extraction from bulk RNA sequencing results, we used XCALIBR (https://github.com/NKI-GCF/xcalibr). Lineage barcodes were extracted from the scRNA-seq data using the CellTag pipeline (https://github.com/morris-lab/BiddyetalWorkflow).<br><br>The raw CGR8 scRNA-seq data was processed using Cell Ranger (v 5.0.1; 10X Genomics). Analysis of scRNA-seq data was further performed using custom code in R (v 4.0.2) and Python (v 3.9.7) (scripts available upon request). Essential packages used in R are topGO (v 2.42.0), velocyto (v 0.6), and Seurat (v 4.1.0), scran (v 1.18.7), circlize (v 0.4.15), clustree (v 0.5.0), igraph (v 1.3.4), dendextend (v 1.16.0), stats (v 3.6.2 and 4.0.2 as indicated), MASS (v 7.3-58.3), tsne (v 0.1-3.1), moments (v 0.14.1), Metrics (v 0.1.4) and dbscan (v 1.1-11). Essential libraries used in Python are numpy (v 1.20.3), pyreadr (v 0.4.4), scipy_cut_tree_balanced (v 1.1), sklearn (v 1.1.1), scipy (v 1.7.1), pandas (v 1.3.4), Pytorch (v 1.11.0), and Optuna (v 2.10.0).<br><br>For analysis of flow cytometry data we used FlowJo (v 10.7.0; Tree Star). For analysis of microscopy images we used ImageJ (v 2.1.0).

For manuscripts utilizing custom algorithms or software that are central to the research but not yet described in published literature, software must be made available to editors and reviewers. We strongly encourage code deposition in a community repository (e.g. GitHub). See the Nature Portfolio guidelines for submitting code & software for further information.

Data

Policy information about availability of data

All manuscripts must include a data availability statement. This statement should provide the following information, where applicable:

- Accession codes, unique identifiers, or web links for publicly available datasets
- A description of any restrictions on data availability
- For clinical datasets or third party data, please ensure that the statement adheres to our policy

The CGR8 scRNA-seq data is available at https://www.ncbi.nlm.nih.gov/geo/ under GSE226169. Other datasets analyzed in the paper are available under GEO accession numbers GSE99915 (MEF), GSE74923 (CD8, L1210), GSE167317 (HSC), GSE237228 (WM989), GSE173958 (pancreatic metastases), GSE148093 (crypts and organoids), GSE140802 (HSPC), GSE243280 (human breast cancer data), GSE182685 (human bone marrow cells and K562). Source data to produce all main figures of the paper are available on Zenodo doi: 10.5281/zenodo.10581737.

Research involving human participants, their data, or biological material

Policy information about studies with human participants or human data. See also policy information about sex, gender (identity/presentation), and sexual orientation and race, ethnicity and racism.

Reporting on sex and gender	N/A
Reporting on race, ethnicity, or other socially relevant groupings	N/A
Population characteristics	N/A
Recruitment	N/A
Ethics oversight	N/A

Note that full information on the approval of the study protocol must also be provided in the manuscript.

Field-specific reporting

Please select the one below that is the best fit for your research. If you are not sure, read the appropriate sections before making your selection.

- [x] Life sciences
- [ ] Behavioural & social sciences
- [ ] Ecological, evolutionary & environmental sciences

For a reference copy of the document with all sections, see nature.com/documents/nr-reporting-summary-flat.pdf

Life sciences study design

All studies must disclose on these points even when the disclosure is negative.

Sample size	No sample sizes were chosen. No method to calculate sample size was used.
Data exclusions	Lineage assignment using cellular barcodes in the CGR8 scRNA-seq data was generated using the CellTag pipeline as indicated above. We used Jaccard similarity index of 70% in barcode expression to call ground truth lineages. Cell lineages with sizes >5 cells were excluded in further analysis, as these cannot occur after the given culture time.
Replication	The experiment to generate lineage-annotated CGR8 scRNA-seq data was only performed once.
Randomization	Randomization was not applicable for this study as no groups were allocated.
Blinding	Blinding was not applicable for this study as no groups were allocated.

Reporting for specific materials, systems and methods

We require information from authors about some types of materials, experimental systems and methods used in many studies. Here, indicate whether each material, system or method listed is relevant to your study. If you are not sure if a list item applies to your research, read the appropriate section before selecting a response.

Materials & experimental systems

n/a	Involved in the study
x	Antibodies
	x Eukaryotic cell lines
x	Palaeontology and archaeology
x	Animals and other organisms
x	Clinical data
x	Dual use research of concern
x	Plants

Methods

n/a	Involved in the study
x	ChIP-seq
	x Flow cytometry
x	MRI-based neuroimaging

Eukaryotic cell lines

Policy information about cell lines and Sex and Gender in Research

Cell line source(s)	In the study only experiments with the CGR8 cell line purchased from ATCC.
Authentication	After purchase from ATCC, no further authentication have been performed.
Mycoplasma contamination	Mycoplasma-negative (bi-annual mycoplasma screening).
Commonly misidentified lines (See ICLAC register)	None

Plants

Seed stocks	N/A
Novel plant genotypes	N/A
Authentication	N/A

Flow Cytometry

Plots

Confirm that:

- [x] The axis labels state the marker and fluorochrome used (e.g. CD4-FITC).
- [x] The axis scales are clearly visible. Include numbers along axes only for bottom left plot of group (a 'group' is an analysis of identical markers).
- [x] All plots are contour plots with outliers or pseudocolor plots.
- [x] A numerical value for number of cells or percentage (with statistics) is provided.

Methodology

Sample preparation	CGR8 cells were transduced with the lentiviral LARRY barcoding library to obtain roughly 1% of GFP+ cells. 72h after infection, live GFP+ cells (live stain 1:500 propidium iodide solution (BioLegend #421301)) were sorted on an FACSAria III (BD) at the Flow Cytometry Core Facility of EPFL into Cellcarrier 96-Ultra 96-well plates (PerkinElmer #6055308).
Instrument	FACSAria III (BD)
Software	FACSDiva (BD) and FlowJo (v 10.7.0; Tree Star).
Cell population abundance	Live single GFP+ cells represented 0.35% of the cell population. Purity of the sorted population was determined using fluorescence microscopy for GFP.

Gating strategy	Cells were gated as single cells using SSC-A/FSC-A gate (to exclude debris), and FSC-H/FSC-W and SSC-H/SSC-W gates to isolate single cells. Single cells were further gated as live cells (PI negative in PI/FSC-A) and GFP+ cells (empty/GFP).

☒ Tick this box to confirm that a figure exemplifying the gating strategy is provided in the Supplementary Information.
